# Epigenetic aging differences between Wichí and Criollos from Argentina: Insights from genomic history and ecology

**DOI:** 10.1093/emph/eoad034

**Published:** 2023-10-16

**Authors:** Vincenzo Iannuzzi, Stefania Sarno, Marco Sazzini, Paolo Abondio, Claudia Sala, Maria Giulia Bacalini, Davide Gentilini, Luciano Calzari, Federica Masciotta, Paolo Garagnani, Gastone Castellani, Edgardo Moretti, Maria Cristina Dasso, Federica Sevini, Zelda Alice Franceschi, Claudio Franceschi, Davide Pettener, Donata Luiselli, Cristina Giuliani

**Affiliations:** Department of Biological, Geological and Environmental Sciences, Laboratory of Molecular Anthropology & Centre for Genome Biology, University of Bologna, Bologna, Italy; Department of Biological, Geological and Environmental Sciences, Laboratory of Molecular Anthropology & Centre for Genome Biology, University of Bologna, Bologna, Italy; Department of Biological, Geological and Environmental Sciences, Laboratory of Molecular Anthropology & Centre for Genome Biology, University of Bologna, Bologna, Italy; Alma Mater Research Institute on Global Challenges and Climate Change (Alma Climate), Interdepartmental Centre, University of Bologna, Bologna, Italy; Department of Cultural Heritage (DBC), University of Bologna, Ravenna Campus, Ravenna, Italy; Department of Medical and Surgical Science (DIMEC), University of Bologna, Bologna, Italy; IRCCS Istituto delle Scienze Neurologiche di Bologna, 40139 Bologna, Italy; Department of Brain and Behavioral Sciences, Università di Pavia, Pavia, Italy; Bioinformatics and Statistical Genomics Unit, Istituto Auxologico Italiano IRCCS, Cusano Milanino, Milan, Italy; Bioinformatics and Statistical Genomics Unit, Istituto Auxologico Italiano IRCCS, Cusano Milanino, Milan, Italy; Department of Statistical Sciences ‘Paolo Fortunati’, Alma Mater Studiorum, University of Bologna, Bologna, Italy; Department of Medical and Surgical Science (DIMEC), University of Bologna, Bologna, Italy; IRCCS Azienda Ospedaliero-Universitaria di Bologna, Bologna, Italy; Department of Medical and Surgical Science (DIMEC), University of Bologna, Bologna, Italy; Facultad de Ciencias Médicas, Universidad Nacional de Córdoba, Instituto de Biología y Medicina Experimental de Cuyo, CCT CONICET, Argentina; Centro de Investigaciones en Antropología Filosófica y Cultural (CIAFIC), Buenos Aires, Argentina; Department of Medical and Surgical Science (DIMEC), University of Bologna, Bologna, Italy; Department of History and Cultures, University of Bologna, Bologna, Italy; Laboratory of Systems Medicine of Healthy Aging and Department of Applied Mathematics, Lobachevsky University, Nizhny Novgorod, Russia; Department of Biological, Geological and Environmental Sciences, Laboratory of Molecular Anthropology & Centre for Genome Biology, University of Bologna, Bologna, Italy; Department of Cultural Heritage (DBC), University of Bologna, Ravenna Campus, Ravenna, Italy; Department of Biological, Geological and Environmental Sciences, Laboratory of Molecular Anthropology & Centre for Genome Biology, University of Bologna, Bologna, Italy

**Keywords:** human aging, epigenetic clocks, genomic history, South America, DNA methylation

## Abstract

**Background and objectives:**

Epigenetic estimators based on DNA methylation levels have emerged as promising biomarkers of human aging. These estimators exhibit natural variations across human groups, but data about indigenous populations remain underrepresented in research. This study aims to investigate differences in epigenetic estimators between two distinct human populations, both residing in the Gran Chaco region of Argentina, the Native-American Wichí, and admixed Criollos who are descendants of intermarriages between Native Americans and the first European colonizers, using a population genetic approach.

**Methodology:**

We analyzed 24 Wichí (mean age: 39.2 ± 12.9 yo) and 24 Criollos (mean age: 41.1 ± 14.0 yo) for DNA methylation levels using the Infinium MethylationEPIC (Illumina) to calculate 16 epigenetic estimators. Additionally, we examined genome-wide genetic variation using the HumanOmniExpress BeadChip (Illumina) to gain insights into the genetic history of these populations.

**Results:**

Our results indicate that Native-American Wichí are epigenetically older compared to Criollos according to five epigenetic estimators. Analyses within the Criollos population reveal that global ancestry does not influence the differences observed, while local (chromosomal) ancestry shows positive associations between specific SNPs located in genomic regions over-represented by Native-American ancestry and measures of epigenetic age acceleration (AgeAccelHannum). Furthermore, we demonstrate that differences in population ecologies also contribute to observed epigenetic differences.

**Conclusions and implications:**

Overall, our study suggests that while the genomic history may partially account for the observed epigenetic differences, non-genetic factors, such as lifestyle and ecological factors, play a substantial role in the variability of epigenetic estimators, thereby contributing to variations in human epigenetic aging.

## INTRODUCTION

Global demographic changes are reshaping age structures across human populations, with aging being a significant risk factor for many chronic diseases. Measuring the rate of aging is thus crucial for extending health span, particularly in diverse human populations, and there is an urgent need to identify factors that increase individual risk of age-related diseases and mortality.

The rate of biological aging showed natural variations among human groups, but to date, a comprehensive description of the aging process in different human populations, especially in indigenous ones, remains limited.

Various measures of biological age aim to capture the process of aging, with epigenetic clocks emerging as the most promising tools capable of distinguishing chronological age from biological age. Epigenetic clocks are based on combinations of DNA methylation (DNAm) levels of CpG sites across the genome, and a substantial body of literature provides compelling evidence about the capability of these biomarkers to capture aspects of biological aging. In general, individuals whose biological age estimates exceed their chronological age are at an increased risk of multiple adverse health outcomes.

**Figure 1.**
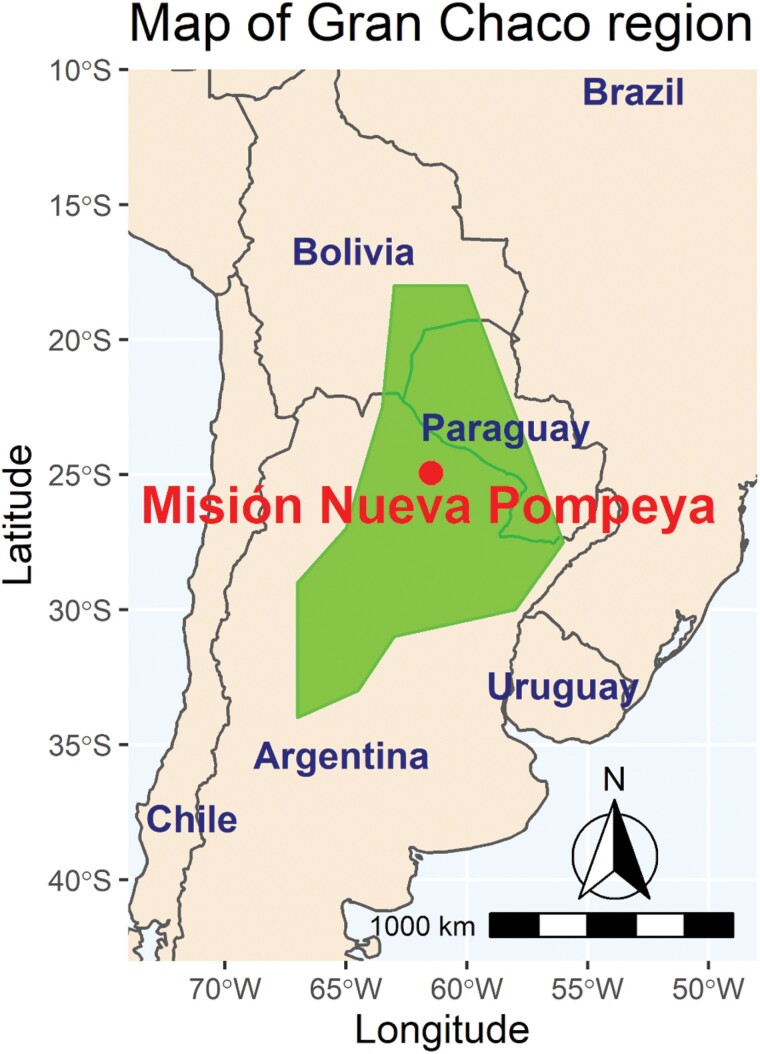
Approximate area of the Gran Chaco region with highlighted Misión Nueva Pompeya where the Wichí and Criollos samples were collected

In this study, we investigate the relationship between the variability of epigenetic estimators and the population genetic structure, by analyzing two distinct communities residing within the village of Mision Nueva Pompeya, in the Gran Chaco region of Argentina (Fig. 1). Within this restricted geographical area, two human populations of different ancestry—the Wichí and the Criollos—cohabit and maintain very different genetic and linguistic profiles, being deeply different also in terms of cultural and social structures, as revealed by ethnographic fieldwork.

The Wichí are indigenous Native-American people speaking a language that belongs to the Matako-Maka family. Recent analyses of genetic and genomic data [1–3] have suggested a prolonged isolation of this indigenous group that, after the initial peopling of the area, was not involved in gene flow events from both the neighboring Andes on the West and the Amazonian regions on the North. Further genetic evaluations also suggested that the Wichí population belongs to the same basal non-Andean lineage of all Amazonians and that they likely descend from one of the first splits within this branch [1]. The Criollos population is instead characterized by a high degree of admixture. This admixture can be attributed to historical intermarriages among various Chaquean groups and the first European male colonizers [3].

In this geographical area, Chagas disease is endemic, with no differences in susceptibility to *Trypanosoma cruzi* (T. cruzi) infection observed between the two groups under consideration [4]. Data indicate that serological prevalence in these areas is more than tenfold higher compared to the average of the country and that disparities were related to habitat rather than to ethnicity [4].

To estimate the discrepancy between chronological and biological ages in these populations we used epigenetic age acceleration measures belonging to the first and second generation of epigenetic clocks. The first-generation clocks, including the widely used Hannum [5] and Horvath clocks [6], are trained on chronological age and applicable to blood as well as 51 human tissues and cell types. The second-generation clocks (*GrimAge* and *PhenoAge*) extend their scope to incorporate information about morbidity and mortality risk [7, 8]. A comprehensive description of epigenetic clocks can be found in recent reviews [9, 10]. Additionally, we employed other biomarkers derived from DNAm levels at CpG sites across the genome to infer an individual’s health status. These biomarkers encompass telomere length [11] and plasmatic components contributing to the *GrimAge* clock [7], offering valuable insights into an individual’s biological aging. For the purposes of this paper, we refer to all these biomarkers collectively as ‘epigenetic estimators’.

These estimators have been applied broadly to study centenarians [12], socioeconomic inequalities, education [13], nutritional status and lifetime stress [14] and conditions including Down’s syndrome [15], Alzheimer’s diseases and cognitive decline [16]. Furthermore, some studies have suggested variations in epigenetic age estimations across different human populations and ethnic groups [17–19]. These differences have been largely attributed to social and environmental health disparities, but in many contexts, these two factors covary with genetic ancestry. Thus, studies that include rigorous analyses of population genomic history and genetic ancestry in different populations are needed. An exemplary study by Gopalan *et al.* [20] addressed the extent to which epigenetic estimators perform consistently across populations with different ancestries, employing accurate methods to reconstruct admixture events. In particular, the study showed that population genetic variation can to some extent influence epigenetic age-prediction algorithms, as a consequence of population-specific patterns at meQTLs (i.e. SNPs associated with DNAm levels at specific CpG sites).

Nevertheless, current research efforts in this area have mainly focused on groups of European ancestry, thus highlighting the urgent need to broaden these investigations to encompass also non-European populations and indigenous groups.

Considering this context, the aims of this study are as follows: (i) to investigate variations in biological aging based on epigenetic estimators between Wichí and Criollos, while also considering data on infection transmitted by the parasite T. cruzi; (ii) to ascertain whether these differences may be attributed to different genetic backgrounds. In this respect, to reduce potential biases we specifically focused on the Criollos, addressing the role of global and local ancestry in determining the observed differences; (iii) to investigate the relative importance of genomic history and of established demographic and environmental factors, aiming to elucidate the underlying drivers of the observed inter-population differences.

## METHODOLOGY

### Study design

**Figure 2.**
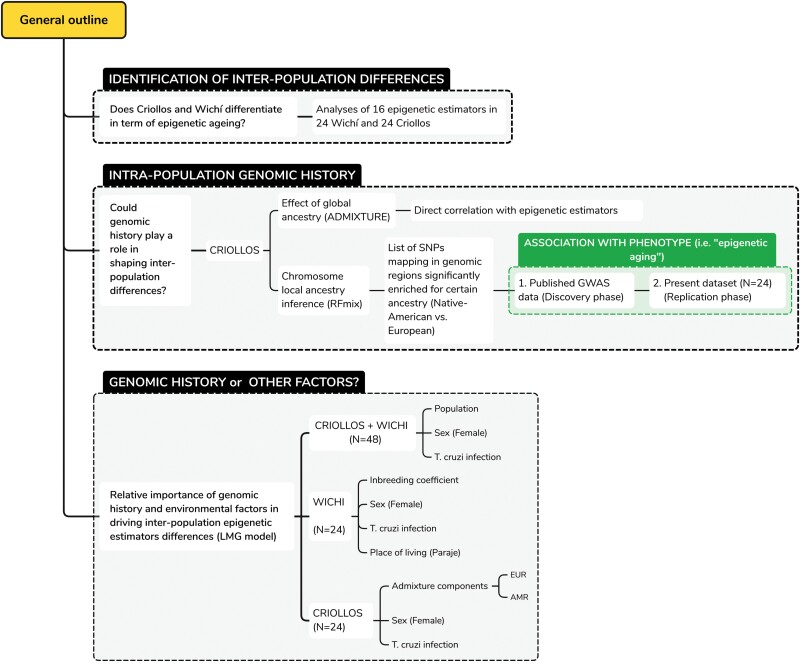
Outline of the analyses performed in the present study

The rationale guiding this study is summarized in Fig. 2 and is based on three major approaches:

1. ‘Analysis of inter-population differences’ (Fig. 2): Initially, we investigated potential differences in biological aging between the Criollos and Wichí populations considering 16 epigenetic estimators, as outlined in Table 1.
2. ‘Intra-population genomic history’ (Fig. 2): Then, we assessed whether the observed differences could be attributed to micro-evolutionary processes. The analyses conducted were designed to align with existing knowledge about the demographic history of the Criollos population. Specifically, we focused on Criollos as it is possible to investigate the extent to which varying ancestries might contribute to inter-population differences in epigenetic estimators within a more homogeneous environmental, ecological and social context. Given that all Criollos come from urban areas, while the majority of Wichì (20/24) reside in rural areas, this approach helped reduce potential confounding effects linked to the place of living and related environmental factors. The analyses were conducted first via global ancestry estimation and then by performing chromosome local ancestry inference. A correlation analysis was performed to assess the potential role of genetic components derived from the global ancestry estimation in influencing the epigenetic estimators. Chromosome local ancestry estimation returned SNPs located within candidate genomic regions enriched for certain ancestry (Native American vs. European). These SNPs were then tested for association with epigenetic estimators (highlighted in green in Fig. 2). Due to the small sample size, the association was first assessed by querying the results from previously published GWAS. Subsequently, the identified signals were validated in our dataset.
3. Lastly, we quantified the extent of genomic history’s impact on inter-population differences in epigenetic estimators. To accomplish this, we compared the relative importance of some factors identified through the previous analyses with that of demographic and environmental factors, including sex, T. cruzi infection and place of living (box ‘Genomic History or Other Factors?’ in Fig. 2).

### Sample description and data generation

We considered 24 individuals for the Wichí group and 24 for the Criollos group. The mean age for the Wichí group was 39.2 ± 12.9 years and for the Criollos group 41.1 ± 14.0 years. For each group, we selected 12 males and 12 females. Fifty percent (12/24, 6 males and 6 females) of Wichí and 50% (12/24, 6 males and 6 females) of Criollos were infected by T. cruzi. We purposely selected the same ratio of infected samples to minimize bias when comparing Wichì and Criollos and when comparing infected individuals to non-infected ones. All the considered Criollos individuals and 4 out of 24 Wichí individuals originated from urban area of Misiòn Nueva Pompeya, whereas 20 out of 24 Wichí individuals were from rural area of Misiòn Nueva Pompeya, mostly residing within the forests. T. cruzi infection was measured through serological tests conducted in 2008 and 2010. Whole blood samples were obtained from venous blood. Subsequently, genomic DNA was extracted from 0.2 ml of whole blood using the QIAamp DNA Blood Midi Kit® (QIAGEN). DNA quantification was performed with a fluorometric dsDNA assay (Quant-iT™ PicoGreen® dsDNA kit, INVITROGEN), and then DNA samples were normalized to a concentration of 22 ng/µl in 45 µl for subsequent bisulfite treatment (EZ DNA Methylation Kits, ZYMO RESEARCH). These samples were analyzed using an epigenome-wide array, the Infinium MethylationEPIC Kit, following the manufacturer’s recommendations. DNA methylation (DNAm) pre-processing analysis was carried out in R 3.3.3 using the *minfi* and *wateRmelon* libraries. The *pfilter* function from *wateRmelon* was applied to filter samples and probes based on bead count and detection *P* values. No samples were filtered out using a significance level of 0.05. Beta values were normalized using the *Dasen* function of the *wateRmelon* package.

A total of 300 ng of DNA from the same samples were also used to genotype ~720 K SNPs distributed throughout the whole genome using the HumanOmniExpress BeadChip (Illumina, San Diego, CA). The resulting genome-wide data comprised 713,014 SNPs for the Wichi and 713,599 SNPs for the Criollos, respectively. A series of quality control steps (QCs) were applied separately to each dataset using the PLINK software version 1.9 [21]. Initially, we verified the reported sex assignments by comparing them with those imputed from the X-chromosome inbreeding coefficient, using the *--check-sex/--impute-sex* functions of the PLINK software. Then, for each population dataset, we retained only autosomal chromosomes and we excluded individuals with a genotyping success rate below 95% (*--mind 0.05*), variants with missing call rates exceeding 5% (*--geno 0.05*) and SNPs showing significant deviations (*P* value < 7.00e−08 after Bonferroni correction) from Hardy–Weinberg equilibrium (*--hwe* option). In addition, we calculated the proportion of alleles shared identical-by-descent (*--genome* function) and excluded one sample for each pair of individuals exhibiting a kinship coefficient (PiHat) higher than 0.25. After QCs and merging procedures, a common dataset for 22 Wichí and 23 Criollos encompassing a shared set of 644,020 autosomal SNPs was retained for further analysis.

The study n° 63/2006/U/TESS was approved on 9 May 2006 by the ethical committees of both the University of Bologna (Azienda Ospedaliero-Universitaria di Bologna—Policlinico Sant’Orsola-Malpighi) and the University Hospital of Maternity and Neonatology, Universidad Nacional de Córdoba, Argentina. Additionally. the Ministry of Health of the province of Chaco provided further authorization to conduct the survey. Participants who voluntarily took part in the study were informed by native-speaking translators and signed a consent form, which was available in both Spanish and Wichí languages.

### Epigenetic estimators and analysis of inter-population differences

To compute epigenetic estimators for the two populations under investigation, we used the online DNAm age calculator (http://dnamage.genetics.ucla.edu) developed by Horvath [6]. We used this calculator to compute 16 measures of epigenetic age adjusted for chronological age (detailed in Table 1) and denoted in the software output with either the *AgeAccel* prefix or the *AdjAge* suffix. These measures are indicative of first-generation epigenetic clocks (Group i in Table 1), second-generation epigenetic clocks (Group ii in Table 1), DNAm-based estimate of telomere length adjusted for age (Group iii in Table 1), which is a well-established biomarker of age [11], and DNAm surrogates of plasma protein components that contribute to the *GrimAge* clock [7] (Group iv in Table 1). DNAm surrogates showed a moderately high correlation (*r* > 0.35) between measured blood levels of the protein and the respective DNA methylation markers [7], but a linear combination of seven DNAm-based surrogate markers of plasma proteins—namely adrenomedullin (ADM), beta-2-microglobulin (B2M), cystatin C (Cystatin C), growth differentiation factor 15 (GDF-15), leptin (Leptin), plasminogen activator inhibitor-1 (PAI-1), and tissue inhibitor metalloproteinases 1 (TIMP-1)—contribute to the *GrimAge* clock [7], a clock which has been associated with all-cause mortality [22]. A description of the epigenetic estimators used in this study is available in Table 1. Following the assessment of the normality distribution assumption for the epigenetic estimators using a Shapiro–Wilk test, we conducted *t*-tests and Wilcoxon tests on the measures of interest contained in the output file to explore possible differences between the two populations. Correction for multiple testing was performed using the Benjamini–Hochberg method (FDR *q*-value < 0.1) [23].

**Table 1.** List of all epigenetic estimators considered in this study.

Epigenetic estimator	Description	Group
AgeAccelerationResidual	DNAm age estimate based on methylation of 353 CpG sites [6]adjusted for chronological age	i
EEAA	DNAm age estimate based on the weighted average of the epigenetic age measure from [5]and three estimated measures of blood cells for cell types that are known to change with age (naïve cytotoxic T cells; exhausted cytotoxic T cells; and plasma B cells) [17]	i
IEAA	DNAm age based on methylation of 353 CpG sites [6] regressed on chronological age and cell count information (naïve CD8 + T cells, exhausted CD8 + T cells, plasmablasts, CD4 + T cells, natural killer cells, monocytes, granulocytes)	i
AgeAccelHannum	DNAm age estimate based on methylation of 71 CpG sites [5] adjusted for chronological age	i
DNAmAgeSkinBloodClockAdjAge	DNAm age estimate based on methylation of 391 CpG sites for human fibroblasts, keratinocytes, buccal cells, endothelial cells, lymphoblastoid cells, skin, blood and saliva samples [6] adjusted for chronological age	i
AgeAccelPheno	DNAm age estimate based on methylation of 513 CpG sites [8] adjusted for chronological age	ii
AgeAccelGrim	DNAm age estimate based on methylation of 1030 CpG sites built on eight DNAm based measures (DNAmADM, DNAmB2M, DNAmCystatinC, DNAmGDF15, DNAmLeptin, DNAmPACKYRS, DNAmPAI1, DNAmTIMP1), chronological age and sex [7]	ii
DNAmTLAdjAge	DNAm-based estimate of telomere length [11] adjusted for chronological age	iii
DNAmB2MAdjAge	DNAm-based prediction of plasma levels of beta-2 microglobulin, a component of major histocompatibility complex class 1 (MHC I) molecular [11], adjusted for chronological age	iv
DNAmADMAdjAge	DNAm-based prediction of plasma levels of adrenomedullin, a vasodilator peptide hormone [7], adjusted for chronological age	iv
DNAmCystatinCAdjAge	DNAm-based prediction of plasma levels of cystatin C or (cystatin 3), formerly called gamma trace, post-gamma-globulin, or neuroendocrine basic polypeptide [7], adjusted for chronological age	iv
DNAmGDF15AdjAge	DNAm-based prediction of plasma levels of GDF-15, growth differentiation factor 15 [7], adjusted for chronological age	iv
DNAmLeptinAdjAge	DNAm-based prediction of plasma levels of leptin, a hormone predominantly present in adipose cells [7], adjusted for chronological age	iv
DNAmPACKYRSAdjAge	DNAm-based prediction of a number of pack of cigarettes during year [7], adjusted for chronological age	iv
DNAmPAI1AdjAge	DNAm-based prediction of plasma levels of plasminogen activator inhibitor antigen type 1 (PAI-1), the major inhibitor of tissue-type plasminogen activator and urokinase plasminogen activator [7], adjusted for chronological age	iv
DNAmTIMP1AdjAge	DNAm-based prediction of plasma levels of TIMP-1 or TIMP metallopeptidase inhibitor 1—a tissue inhibitor of metallo-proteinases [7], adjusted for chronological age	iv

Group i: first-generation epigenetic clocks; Group ii: second-generation epigenetic clocks; Group iii: DNAm-based estimate of telomere length adjusted for chronological age; Group iv: DNAm surrogates of components that contribute to GrimAge clock all adjusted for chronological age.

Finally, a correlation matrix among the 16 epigenetic estimators was generated using the Spearman coefficient (Supplementary Fig. S1). Two epigenetic estimators were considered independent when the Spearman’s correlation coefficient was not significantly different from zero (*P* value < 0.01).

### Inbreeding analysis and association with epigenetic estimators in Wichí

Previous studies [1] have suggested that the Wichí population has long inhabited the Gran-Chaco region and has maintained genetic isolation from both neighboring Andean populations on the West and Amazonian groups on the North. At this perspective, it is reasonable to hypothesize that the genomic history of Wichí may have been characterized by kinship and inbreeding. To assess kinship between all pairs of Wichí individuals and to estimate individual inbreeding coefficients, we employed the REAP software [24]. We ran the REAP software using the allele frequency file and the individual ancestry proportion file obtained from ADMIXTURE output for the Wichí population. In particular, the estimation of these coefficients was based on four continental ancestry components (namely Asian, African, European and Native-American ancestry).

After having checked the normality distribution assumption for the epigenetic estimators in the Wichí population using a Shapiro–Wilk test, we conducted Pearson and Kendall tests to explore the association between inbreeding and epigenetic estimators. Additionally, for the purpose of comparing kinship coefficients (computed for pairs of individuals) with epigenetic estimators (computed for each individual), we introduced a ‘delta’ measure, defined as the difference between the values of the epigenetic estimators of two individuals. If kinship affects the epigenetic signals, the more two individuals are related, the lower ‘delta’ is expected.

### Analyses in Criollos

#### Global ancestry analysis and association with epigenetic estimators in Criollos

The common dataset consisting of 644 020 autosomal SNPs shared between Wichí and Criollos was merged with publicly available genome-wide data for populations of African, East Asian, European and Native-American ancestry. These reference datasets were sourced from the Human Genome Diversity Project, the 1000 Genomes Project, and literature-based data on Native-American groups from Central and South America (Supplementary Table S1). The same QCs described in Section 2 were performed on these reference datasets, by further removing ambiguous A/T and C/G polymorphisms to avoid strand-flipping issues during the merging procedure with our ‘local’ set. After the merging process, we obtained an ‘extended’ dataset comprising a total of 1349 individuals from 36 population groups, genotyped for a set of 349 099 common SNPs. This ‘extended’ dataset was pruned for genotype-based analyses by removing SNPs in high linkage disequilibrium (*r*^2^ > 0.2) within a sliding window of 50 SNPs, advancing by 5 SNPs at the time (*--indep-pairwise 50 5 0.2* command in PLINK). After pruning a total of 132 838 SNPs were retained.

To infer the genetic ancestry composition of the analyzed populations, we applied the unsupervised clustering algorithm of the software ADMIXTURE [25] to the pruned extended dataset, by testing hypothetical ancestral populations (*K*) ranging from 2 through 12. We performed 10 independent runs for each value of *K* with different random seeds, and then we retained the runs with the highest log-likelihood values to generate the final admixture plot. We used the cross-validation (CV) errors associated with the given replicates of *K* to identify the number of *K* that provided the best fit to the data.

The global ancestry components inferred with ADMIXTURE for Wichí and Criollos at both the continental level of *K* = 4 and the best value of *K* = 8 (according to CV errors), were correlated with the epigenetic estimators by using the *cor.test* function available in the software R [26]. Before performing the correlation analysis, we assessed the normality or significant deviation from normality in the distribution of both genetic and epigenetic variables by using the Shapiro–Wilk normality test (implemented in the R software function *shapiro.test*).

#### Local ancestry inference and association with epigenetic estimators in Criollos

The local ancestry composition of our target admixed population (i.e. Criollos) was estimated using the RFMix (v.1.5.4) software [27]. We assembled the reference panel of putative source populations by initially selecting 83 individuals from the extended dataset who displayed Native-American ancestry exceeding 0.99 at the considered *K* = 4 continental level of admixture. The individuals selected for the Native-American reference panel specifically encompassed 8 Ashaninka, 6 Cashibo, 6 Huambisa, 11 Shipibo, 21 Yanesha, 4 Bolivia_Aymara, 14 Titicaca_Aymara, 9 Titicaca_Quechua and 4 Titicaca_Uros. Then, to reduce potential biases in the inference procedure arising from different reference panel sizes, we randomly selected an identical number of 83 individuals from the European (CEU—Utah residents with European ancestry from the CEPH collection; GBR—British from England and Scotland; IBS—Iberian populations in Spain; TSI—Tuscan in Italy), African (ESN—Esan in Nigeria; GWD—Gambian in Western Division; LWK—Luhya in Webuye, Kenya; MSL—Mende in Sierra Leone; YRI—Yoruba in Ibadan, Nigeria) and East Asian (CDX—Chinese Dai in Xishuangbanna, China; CHB—Han Chinese in Beijing, China; CHS—Han Chinese South, China; JPT—Japanese in Tokyo, Japan; KHV—Kinh in Ho Chi Minh City, Vietnam) populations of the 1000 Genomes Project [28], to be used as proxies for the corresponding reference ancestries. The unpruned dataset of 645 288 SNPs resulting from the merging process was phased with SHAPEIT (v2.r790) [29] using the 1000 Genomes Phase 3 reference panel and genetic maps. The phased dataset was then transformed into the necessary RFMix input files, following the pipeline reported by Martin *et al.* [30]. We ran RFMix in ‘*PopPhased*’ mode, using a minimum window size of 0.2 cM (*-w 0.2* flag), performing one expectation–minimization iteration (-*em 1* flag) with a node size of 5 (*–n 5* flag), by further maintaining the reference panel after the initial inference step (i.e. using the *--use-reference-panels-in-EM* flag) and saving the forward-backward posterior probabilities (using the *--forward-backward* flag). Finally, we applied a posterior probability threshold of > 0.9 to assign a given allele at a SNP for each individual haplotype to a specific ancestry, otherwise, it remained with an unknown/undefined ancestry assignment.

The results of the local ancestry inference analysis were used to identify genomic regions that displayed significant contributions from one of the ancestral source populations, compared to what would be expected based on the genome-wide average ancestry proportion of the entire population [31]. Considering the generally low proportions of African (<5%) and Asian (<1%) ancestry detected in the Criollos population based on both global and local ancestry estimates, for this analysis we concentrate exclusively on the main Native-American and European components of admixture. Additionally, we excluded from the calculations two Criollos individuals who showed extreme proportions of these ancestries. Ancestry-specific *Z*-scores were calculated for each SNP as the number of standard deviations above or below the genome-wide ancestry average, using the formula: *Z*_anc_ = (*f*_anc_ − *μ*_anc_)/*σ*_anc_. Corresponding *P* values were computed using *Z* distribution [31]. Ancestry-specific regions were finally defined as windows of consecutive SNPs showing significant *Z*-score results (specifically |*Z*| > 3).

SNPs located within the genomic regions identified by the local ancestry analysis were then merged with a reference list of SNPs obtained from a previous GWAS [32], with the aim to identify signals of association with epigenetic estimators (‘discovery phase’). This reference list encompasses genome-wide associations with epigenetic estimators for 137 significant SNPs, resulting from a trans-ethnic meta-analysis on 34 710 individuals of European ancestry and 6195 African Americans. Only SNPs retrieved from the GWAS were considered for validation in our cohort of Criollos (‘replication phase’). The significance in the replication phase was assessed using nominal *P*-values < 0.01.

We described the genomic regions identified in both the discovery and replication phases, and we extracted the *P* values of all SNPs located within these identified regions from the supplementary lists provided by McCartney *et al.* [32]. These lists report the statistics referred to the genome-wide association with epigenetic estimators for approximately 6 million SNPs.

### Relative importance of predictors in influencing the variance of epigenetic estimators

**Figure 3.**
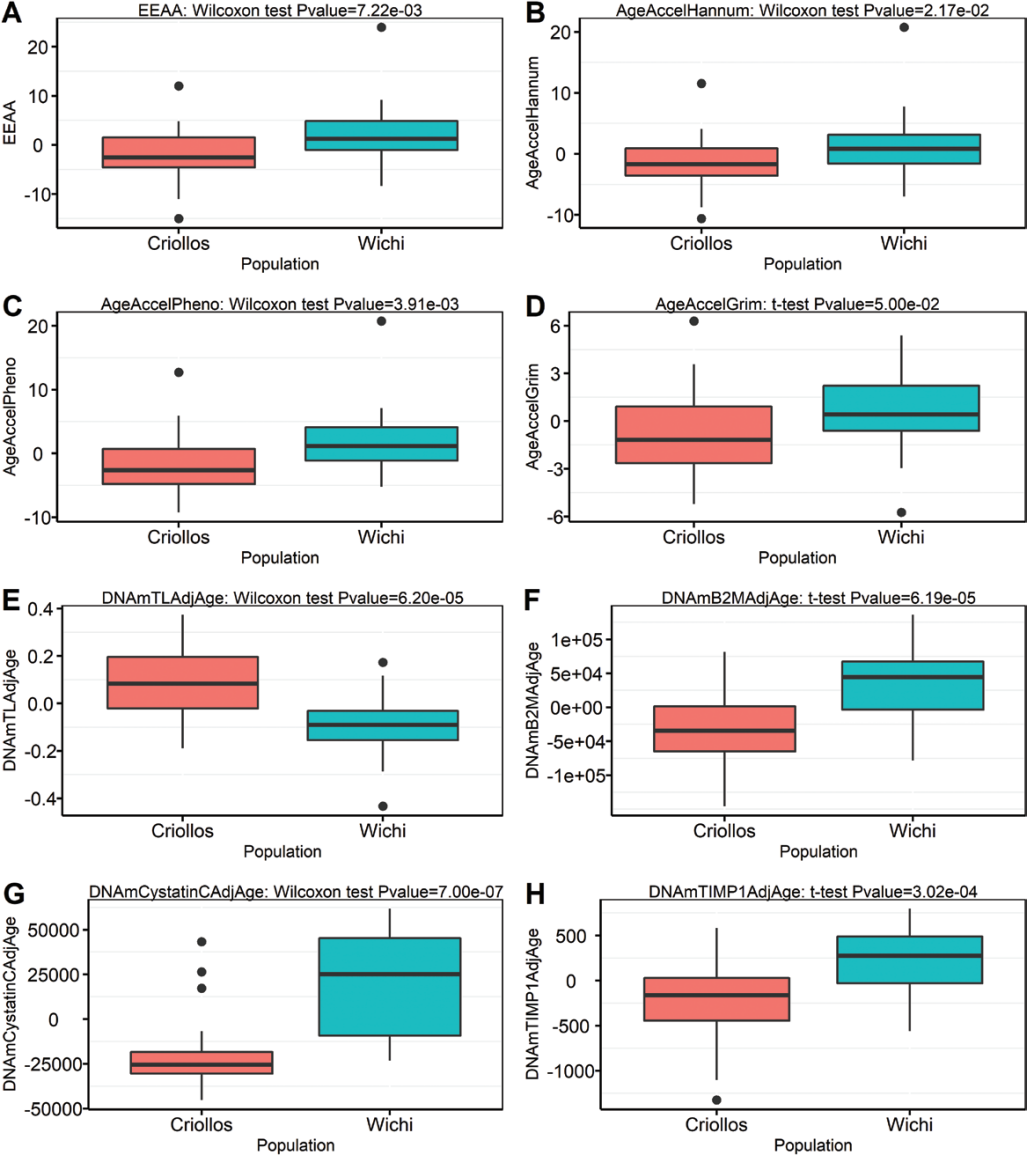
Boxplots for the epigenetic estimators that showed significant differences between the two analyzed populations

For each significant epigenetic estimator identified in the inter-population differences analysis (Fig. 3), we then estimated three linear regression models to assess the influence of genomic history, along with that of known demographic or environmental factors, on the observed inter-population differences. The first model considered Wichí and Criollos together, the second considered only Wichí, and the third considered only Criollos.

In the model considering both Wichí and Criollos together, we incorporated the following predictors: ‘Pop’, set to 1 for Wichí individuals and 0 for Criollos; ‘Female’, set to 1 for females and 0 for males; and ‘T.cruzi’, set to 1 for individuals infected by *T. cruzi* and 0 for those who are not.

In the model considering only Wichí, the following predictors were included: ‘Inbreed’, denoting the inbreeding coefficient as estimated by REAP software; ‘Paraje’ (place of living), set to 1 for individuals living in urban areas and 0 for individuals living in rural areas (the division into urban and rural areas was performed according to the Fig. 1A of [4] as the individuals come from *Güemes, Polenom, Atento, Pozo del Toba and Pozo del Sapo, Rosa Supaz, and Araujo*); ‘Female’; and ‘T.cruzi’.

In the model considering only Criollos, the following predictors were included: ‘EUR’ (European ancestry proportion); ‘AMR’ (Native-American ancestry proportion); ‘Female’ and ‘T.cruzi’. The ancestry proportions were those obtained from ADMIXTURE results at *K* = 4 (i.e. considering the main continental level of admixture). However, given the generally low contributions from the Asian and African components to the population, we decided not to include these two ancestry proportions in the model to reduce potential multicollinearity issues.

To assess the impact of these factors on the variance of epigenetic estimators, we computed the relative importance of the predictors employing the Lindemann, Merenda and Gold (LMG) statistic [33] and using the *relaimpo* package of the R software [34]. In each of the three models, the predictor with the highest LMG value was recognized as the most important factor influencing the variance of an epigenetic estimator. Furthermore, we assessed the percentage of the total variance of each epigenetic estimator explained by the most important predictor as the product of the LMG value and the *R*-squared value of each model.

## RESULTS

### Differences in epigenetic estimators between Wichí and Criollos

Epigenetic estimators were calculated for both Wichí and Criollos populations considering DNA methylation profiles obtained from the Illumina Infinium EPIC platform and applying a previously established method [6]. Initially, we validated the accuracy of the DNAm age estimates in predicting the chronological age within our study populations. We consistently found that the correlation between DNA methylation-age estimates (Horvath, Hannum, Pheno, GrimAge and Skin Blood clock) and chronological age exceeded 0.9 for Criollos. Instead, lower correlations were observed for Wichì (ranging between 0.7 and 0.8) except for *AgeAccelGrim* estimator, which exhibited a correlation value of 0.97 (see Supplementary File 1 for additional details).

For our subsequent analyses, we considered a total of 16 epigenetic estimators: 5 first-generation epigenetic clocks (Group i), 2 second-generation epigenetic clocks (Group ii), 1 DNAm-based estimate of telomere length corrected for age (Group iii) and 8 DNAm surrogates of plasma protein components that contribute to the *GrimAge* clock, all adjusted for age (Group iv). A detailed description of these groups can be found in Table 1.

We conducted *t*-tests and Wilcoxon tests to investigate differences in epigenetic estimators between the two populations, and the resulting *P*-values were corrected using the Benjamini–Hochberg method. As reported in Fig. 3, we found that Wichí exhibit significantly higher levels than Criollos in two first-generation epigenetic clocks (*EEAA, AgeAccelHannum*), two second-generation epigenetic clocks *(AgeAccelPheno* and *AgeAccelGrim)*, and significantly lower levels in the estimated telomere length represented by *DNAmTLAdjAge* (nominal *P* values < 0.01). Overall, these results suggest that Wichí are epigenetically older than Criollos.

Furthermore, Wichí individuals showed significantly higher levels in three DNAm surrogates of plasmatic components, specifically *DNAmB2MAdjAge, DNAmCystatinCAdjAge* and *DNAmTIMP1AdjAge* (nominal *P* values < 0.01).

All the mentioned epigenetic estimators remained statistically significant after multiple test correction (FDR < 0.1). Notably, the discrepancy between epigenetic age and chronological age, measured using the median of the four epigenetic clocks (*EEAA, AgeAccelHannum, AgeAccelPheno, AgeAccelGrim)*, consistently showed positive values in Wichí (1.26, 0.87, 1.19, 0.42, respectively) and negative values in Criollos (−2.55, −1.67, −2.62, −1.16, respectively).

The correlation matrix among the 16 measures (Supplementary Fig. S1) identified four distinct groups (as reported in Fig. 3) of epigenetic estimators independent of each other, though those within the same group were correlated. The first group comprises *AgeAccelPheno, EEAA, AgeAccelHannum* and *DNAmTLAdjAge*; the second group includes *AgeAccelGrim* and *DNAmTIMP1AdjAge*; the third and fourth groups consist of *DNAmCystatinCAdjAge* and *DNAmB2MAdjAge*, respectively. Consequently, these results indicate that four independent signals detected population differences.

All the before-mentioned results collectively suggest that Wichí, representing the Native American group, exhibit signs of being epigenetically older than admixed Criollos. Accordingly, also predicted epigenetic age was higher than the observed chronological age in Wichí, while the opposite trend was observed for Criollos. We did not include the place of living as a covariate in our analysis because, in our study design, the place of living (urban vs. rural) covaries with population groups (Criollos vs. Wichì).

Furthermore, none of the analyzed epigenetic estimators were significantly influenced by T. cruzi infection after correcting for multiple testing. Only *DNAmCystatinCAdjAge* displayed marginal differentiation in Criollos, with a nominal *P* value < 0.05 (see Supplementary File 2).

Given the long-term isolation characteristic of the Wichí population [1], we also explored the role of kinship and inbreeding in influencing significant epigenetic signals. However, when we assessed the correlation of inbreeding and kinship coefficients with the eight epigenetic estimators, no significant results (FDR < 0.01) were observed.

### Epigenetic estimators and ancestry proportions in Criollos

Since historical data suggested that Criollos constitute an admixed population, we explored whether the eight epigenetic estimators that differentiated Criollos from Wichí might be influenced by different genetic ancestry components.

To investigate this, we initially inferred global ancestry proportions using ADMIXTURE, by testing 2 through 12 genetic components. The best predictive accuracy, based on cross-validation (CV) errors, was achieved when considering eight ancestral groups (*K* = 8). Comprehensive details on all the admixture analyses are reported in Supplementary File 3. Briefly, the major continental ancestries were distinguished at *K* = 4, delineating the main Native-American, European, African and Asian genetic components. At the best predictive value of *K* = 8, the admixture analysis confirmed the results from previous studies [1], by identifying highly specific Native American genetic ancestry fractions.

We then assessed the correlation between ancestry components and all the 16 epigenetic estimators analyzed, in the admixed Criollos group. Our investigation did not reveal any associations between the genetic components (both at *K* = 4 and *K* = 8) and the eight epigenetic estimators that were found to differentiate Criollos from Wichí. This result suggests that global ancestry is not a primary driver of the observed epigenetic differences between the two populations. For detailed information regarding the associations in all the 16 considered epigenetic estimators, refer to Supplementary File 3.

Chromosome local ancestry inference was then performed to explore if genomic regions enriched for particular ancestries (Native American vs. European) could be responsible for the observed differences. We inferred chromosome local ancestry estimates for each individual of the Criollos admixed population with RFmix using four putative source groups, informative of the main Native-American, European, African and Asian continental genetic components (Supplementary Fig. S2). The obtained chromosome local ancestry patterns were subsequently used to identify genomic regions exhibiting significant deviations (i.e. over- or under-representation) in local ancestry-specific assignment compared to the average ancestry proportion of the entire population (see ‘Methodology’ section for details). In particular, single locus ancestry-specific *Z*-scores were calculated for the Native American and European components, given the considerably low proportions of African and Asian ancestries overall observed in Criollos.

**Figure 4.**
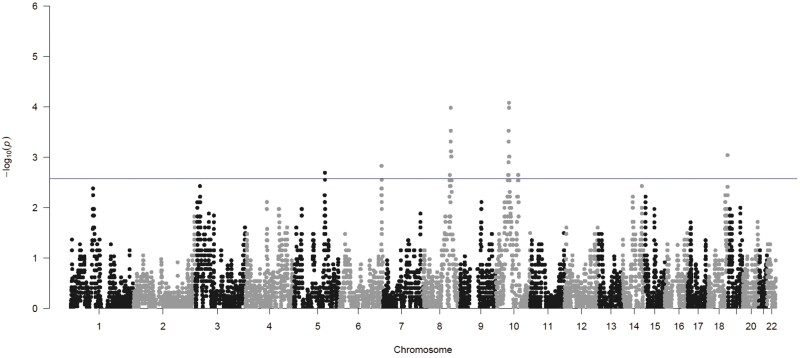
Manhattan plot showing regions with significant over-representation of Native-American ancestry across the genome. Ancestry-specific *Z*-scores were calculated for each SNP as the number of standard deviations above or below the genome-wide ancestry average, and corresponding *P*-values were computed using the *Z* distribution. The *P*-value threshold corresponding to the considered statistical level of significance for |*Z*| > 3 is highlighted with the blue line

Our analysis identified significant signals for 14 genomic regions (Supplementary Table S2), among which the 2 most significant ones were chr8:108647423-111991956 (*Z*-Max = 3.88, *P* value = 1.05e−04) and chr10:48318619-53411242 (*Z*-Max = 3.93, *P* value = 8.35e−05). Interestingly, both of these regions revealed a significant over-representation of Native-American ancestry (Fig. 4 and Supplementary Table S2). Furthermore, partially overlapping sub-regions within these same chromosomal segments also displayed an under-representation of European ancestry (Supplementary Table S2).

We then scanned published GWAS studies on epigenetic estimators [32] to identify significant SNPs located within the genomic regions pinpointed by the local ancestry analysis. To reduce the risk of false-positive associations, we decided to first examine available published data (Discovery Phase), given the relatively small sample size of our cohort. Remarkably, we identified one SNP, named rs4838595_C, mapping in the ARHGAP22 gene and located within the detected chr10:48318619-53411242 region, which exhibited a significant association with the *AgeAccelHannum* epigenetic clock in a trans-ancestry meta-analysis (*P* value = 7.26e−11) [32]. Specifically, McCartney *et al.* [32] found that the Native American ancestral C allele produces a 0.29-year increase in the gap between biological and chronological age (Table 2), thus suggesting a potential deleterious effect of this allele on epigenetic aging.

**Table 2.** List of SNPs located within the chr10:48318619-53411242 window enriched for Native American ancestry that showed significant association with the Hannum epigenetic clock (*AgeAccelHannum*) in the GWAS study by McCartney *et al.* [32].

SNP	chr	pos	Criollos Native-American enriched allele	A1	A2	Freq A1	Effect A1	SE	*P*	*N*
rs2853838	10	48431110	**G**	T	G	0.26	−0.01	0.04	3.89e−03	36 401
rs2853840	10	48442146	**C**	T	C	0.36	−0.05	0.03	8.38e−03	32 961
rs12249222	10	49406469	**C**	C	T	0.69	−0.13	0.03	3.41e−03	36 402
rs1864345	10	49420008	**T**	T	C	0.72	−0.16	0.04	3.83e−04	35 829
rs7068878	10	49420462	**A**	A	C	0.73	−0.16	0.03	1.52e−04	33 536
rs2698761	10	49591718	**C**	C	T	0.45	0.07	0.03	5.09e−03	36 751
** rs4838595	10	49675247	**C**	C	T	0.86	0.29	0.03	7.26e−11	37 244
rs10857580	10	49686384	**C**	C	A	0.86	0.25	0.03	4.10e−08	33 886
rs1077960	10	49698113	**T**	T	G	0.87	0.24	0.03	2.04e−06	36 735
** rs4838608	10	49707942	**A**	A	G	0.85	0.13	0.03	8.00e−04	36 402
** rs4838609	10	49713830	**T**	T	G	0.85	0.12	0.03	1.83e−03	36 398
** rs7896115	10	49730982	**A**	G	A	0.10	−0.13	0.03	7.05e−03	34 381
rs11101371	10	49735098	**A**	A	G	0.80	0.08	0.04	3.95e−03	34 453
rs10776612	10	49735563	**C**	C	T	0.53	0.01	0.03	5.15e−03	37 322
rs2289808	10	49790972	**T**	C	T	0.73	0.12	0.04	6.43e−03	34 451
rs1445164	10	49800427	**G**	G	A	0.90	0.19	0.05	5.81e−04	34 136
rs883017	10	50217438	**C**	T	C	0.45	0.07	0.02	8.44e−03	34 450
rs9804403	10	50262144	**T**	T	C	0.66	−0.08	0.02	2.81e−03	37 216
rs12255055	10	50318484	**G**	G	A	0.57	−0.10	0.03	6.23e−03	36 746
rs7086360	10	50615806	**G**	G	A	0.57	0.11	0.03	1.39e−03	36 750
rs2983362	10	52325713	**C**	T	C	0.16	−0.08	0.04	3.64e−03	34 380
rs10996325	10	53009688	**G**	G	A	0.92	−0.10	0.06	8.98e−03	37 142
rs10823041	10	53223446	**G**	G	A	0.62	−0.10	0.03	6.90e−03	34 455
rs10508942	10	53240022	**G**	G	A	0.55	0.09	0.03	8.82e−03	36 400
rs7077665	10	53365208	**G**	G	A	0.75	−0.05	0.05	8.36e−03	33 796

Only SNPs with a *P* value < 0.01 are reported, along with the corresponding summary statistics of the trans-ancestry meta-analysis. The Native-American enriched allele observed in the Criollos population is also specified. Different color lines are used to indicate SNPs located within the same LD area. The SNPs specifically discussed in the text are indicated with **.

Subsequently, we performed an association analysis between all the SNPs located within this genomic region and the *AgeAccelHannum* epigenetic clock by considering our cohort, which comprises the 24 Criollos (Replication Phase). We found that the Native-American ancestral alleles of three SNPs (rs4838608_A, rs4838609_T, rs7896115_A), which are situated in close proximity to the previously identified rs4838595 and belong to the same linkage disequilibrium area, significantly associate with *AgeAccelHannum* epigenetic clock (nominal *P*-value = 1.70e−03). Detailed data regarding the genomic region surrounding the rs4838595 SNP, as retrieved from McCartney *et. al.*[32], are reported in Table 2.

### Modeling predictors of the epigenetic estimators

**Figure 5.**
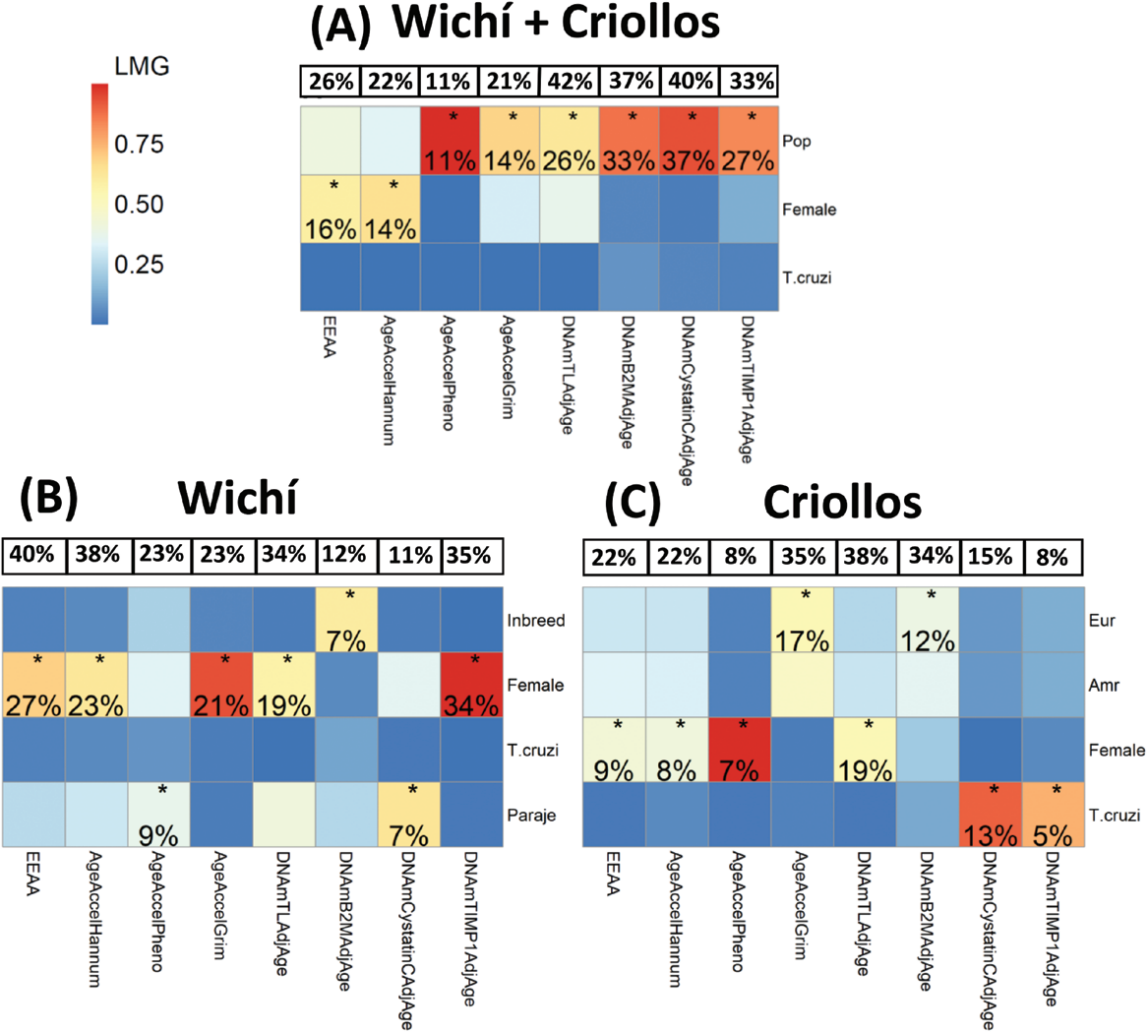
The relative importance of predictors, computed through the LMG statistic, in influencing the variance of the eight epigenetic estimators that differentiate Wichí and Criollos. The most important predictor is indicated with an asterisk (*). The percentage of the total variability of each epigenetic estimator explained by the most important predictor is reported. The percentage of the total variability (*R*-squared) explained by the models is reported at the top of each heatmap. (A) LMG values of predictors considered in the model that was estimated for Wichí and Criollos together. (B) LMG values of predictors considered in the model that was estimated only for Wichí. (C) LMG values of predictors considered in the model that were estimated only for Criollos

Since the previous analyses showed that only one measure of age acceleration (*AgeAccelHannum*) might be in some way influenced by the population’s genomic history, our subsequent analyses aimed to assess the impact of known demographic and environmental factors on the variability of the eight epigenetic estimators that were found to differentiate Wichì and Criollos (Fig. 2). To achieve this, we employed the following approach (i) for each epigenetic estimator, we constructed three linear regression models: one for Wichí and Criollos together, one only for Wichí, and one only for Criollos alone; (ii) we then calculated the normalized LMG statistic to establish the relative importance of the predictor variables; and (iii) we assessed the percentage of the total variance of each epigenetic estimator that could be explained by the most important predictors (Fig. 5).

The regression analysis involving Wichí and Criollos revealed that the variable ‘Pop’ emerged as the most important predictor explaining the variability of *AgeAccelPheno, AgeAccelGrim, DNAmTLAdjAge, DNAmB2MAdjAge, DNAmCystatinCAdjAge* and *DNAmTIIMP1AdjAge* (Fig. 5A), thus indicating the significance of population as a major driver of the differences observed within the communities living in the Gran Chaco of Argentina.

In the regression analysis focusing solely on Wichí, the inbreeding coefficient (‘Inbreed’) emerged as the most important predictor for *DNAmB2MAdjAge* (Fig. 5B). Additionally, the variable ‘Paraje’, which indicates the place of living, appeared the most important variable in explaining the epigenetic variability observed in *AgeAccelPheno* and *DNAmCystatinCAdjAge*, thus suggesting that environmental factors linked to the place of living may contribute to the observed differences.

In the regression analysis dedicated exclusively to Criollos (Fig. 5C), the ‘T.cruzi’ emerged as the most important predictor for *DNAmCystatinCAdjAge* and *DNAmTIMP1AdjAge*. This seems to suggest that infections may modulate changes in *DNAmCystatinCAdjAge* and *DNAmTIIMP1AdjAge*. On the other hand, *DNAmB2MAdjAge* and *AgeAccelGrim* were found to be more influenced by the genomic structure of the population.

As expected, all models consistently highlighted a major influence of sex on the variability of several epigenetic estimators.

Finally, in Fig. 5, we have also reported the percentage of the total variance for each epigenetic estimator that is explained by the most important predictor (indicated with an asterisk). Despite identifying some factors as best predictors, the overall findings indicated that these factors only explain a relatively small percentage of the total variability (less than 10%) in the considered epigenetic estimators. This result, therefore, suggests the potential presence of other unobserved factors that may play a significant role in explaining the observed epigenetic variability.

## DISCUSSION

In this paper, we have proposed an integrated approach that combines a detailed analysis of biological aging measured through DNAm levels (epigenetic clocks and related measures), with the analysis of the evolutionary history of two human groups from the Gran Chaco region of Argentina, which are characterized by different genomic histories: the Wichí, a Native-American population and the Criollos, an admixed population living in the same geographical area. By calculating 16 epigenetic estimators in both Wichì and Criollos, we described for the first time differences between the two populations. Specifically, we found that Native American Wichí appears to be epigenetically older than admixed Criollos according to four epigenetic clocks (*EEAA, AgeAccelHannum, AgeAccelPheno, AgeAccelGrim*) and estimated telomere length (*DNAmTLAdjAge*). Additionally, we identified significant differences in three DNAm surrogates of plasmatic components (*DNAmB2MAdjAge, DNAmCystatinCAdjAge* and *DNAmTIMP1AdjAge*).

These differences we describe can be interpreted on the basis of two possible assumptions:

(1) the association between epigenetic clocks and final outcomes (such as biological aging, all-cause mortality, morbidity, etc.) varies across different human populations;
(2) the association between epigenetic clocks and the final outcomes is consistent across all human groups.

In the first scenario, interpreting the biological significance of the differences in epigenetic clocks observed between Wichì and Criollos becomes complicated, as we do not know whether epigenetic clocks are indeed measuring aging or other biological aspects (e.g. cell differentiation). Additionally, the lack of data about the association with final outcomes opens multiple interpretations because what may appear as a health detriment in one population could potentially be a health enhancer in another (inflammation is an example).

In the second scenario, population-level data may help elucidate some of the observed differences. Some hypotheses are presented below.

Regarding healthcare access, both Wichí and Criollos have access to hospitals and healthcare facilities. However, both populations may also follow, to some extent, traditional treatments involving the use of local plants. Therefore, the absence of accurate data on healthcare practices makes it challenging to draw specific interpretations on this basis.

Nutritional status may play a significant role [35]. While differences in body mass index between Wichí and Criollos are not easy to define due to the often partial data availability, a recent study [36] showed that a substantial percentage—approximately 64,8%—of Wichì adults living in the village of Misión Nueva Pompeya are overweight (34%) or obese (30,8%). Anthropometric analyses also highlighted that Wichí of Misión Nueva Pompeya exhibit short stature combined with central obesity, and previous data in this area showed that these characteristics occur especially among young individuals who experienced forms of early malnutrition. These findings likely reflect the fact that Wichì communities are undergoing a nutritional transition characterized by shifts in lifestyle and dietary habits towards processed foods [36]. Moreover, this nutritional transition aligns with changes in their subsistence patterns, transitioning from a hunter-gatherer to a more sedentary lifestyle. These factors could potentially contribute to some of the observed differences, such as those detected for the *AgeAccelPheno* clock. The *AgeAccelPheno* clock is known to be influenced by environmental factors such as physical exercise and dietary habits [8]. Furthermore, twin and pedigree-based studies have estimated its heritability at approximately 33% [37], which is lower compared to other epigenetic clocks.

Another crucial factor that could contribute to the observed differences is the contrasting living environments between Wichi and Criollos. The majority of Wichí individuals considered in this study indeed reside in rural areas, while all of the considered Criollos are from urban areas.

During the time of biological sample collection, access to drinking water was unavailable to both Wichì and Criollos. Only in very recent years, urban areas have seen improvements in this regard, with the construction of an aqueduct ensuring access to potable water, while rural areas continue to lack this access. Moreover, in Misión Nueva Pompeya, Criollos typically reside in houses equipped with sanitation facilities, whereas both Wichí and Criollos living in rural areas do not have access to sanitation.

In the entire Gran Chaco area, the presence of vectors is much higher in rural villages where houses are typically constructed with adobe and straw roofs thus facilitating vector colonization, compared to urban houses which are built with brick instead of adobe [4]. It is also worth noting that many Wichí adults were probably born in rural houses and only moved to the urban area following the establishment of the Wichí community in Misión Nueva Pompeya during the 1980s (see Ref. [4] for more details). These factors may contribute to some of the differences we are observing, particularly in measures like *EEAA.* The extrinsic epigenetic age acceleration (*EEAA*) indeed correlates with *AgeAccelHannum*, as indicated in Ref. [38], thus capturing a similar aspect of aging. It is constructed using Hannum’s clock and incorporates changes in cell composition by applying a weighted average of age-associated cell counts. Thus, *EEAA* is particularly influenced by age-related alterations in blood cell composition, which can be interpreted as an indicator of immune system aging. Our results—showing significantly higher *EEAA* values in native Wichí—are consistent with recent data from Horvath and colleagues, who reported a higher extrinsic aging rate in the Tsimané, an indigenous population from Bolivia [17]. The authors suggested that this result may be attributed to increased rates of inflammation resulting from recurrent bacterial, viral and parasitic infections [4]. This is noteworthy because numerous studies have emphasized the substantial impact of different ecological settings on epigenetic clocks and on DNA methylation data. For example, a previous study conducted in a small-scale farming society in the Congo Basin showed that epigenetic age acceleration is influenced by early life experiences. In particular, the authors demonstrated that a high burden of infectious diseases led to an increased energy demand, which in turn affects epigenetic age acceleration [39]. Furthermore, Fagny *et al.* [40] showed that in Central Africa, foragers and agriculturalists residing in rainforests experience greater epigenetic age acceleration compared to populations residing in urban settings [40].

Differences between Wichí and Criollos have also been observed in *DNAmTLadjAge*, a predictor of telomere length, with Native-American Wichí showing lower values than admixed Criollos. A negative value of *DNAmTLadjAge* indicates that the estimated telomere length is shorter than expected based on age, while a positive value indicates the opposite. Telomere shortening has been described in populations experiencing extreme stress conditions, such as Indian indigenous people like the Sahariya [41]. However, *DNAmTLAdjAge* not only reflects the replicative history of cells but is also associated with age-related phenotypes [11].

The analysis of micro-evolutionary dynamics in Criollos suggested that the genomic history of these populations may have, at least in part, influenced certain epigenetic estimators, likely contributing to the observed variation. Overall, the admixture analysis performed on Criollos revealed that their genomes consist of an almost 55 mixture of Native-American and European ancestry fractions, which is consistent with historical evidence of continental admixture in this population group. Here, we demonstrated that global ancestry, calculated at both *K* = 4 (continental level) and *K* = 8 (best CV level), did not associate with any of the eight epigenetic estimators that differentiate the two populations. On the other hand, chromosome local ancestry inference identified a region on chromosome 10, in which Criollos show an excess of Native-American ancestry tracts, partially overlapped by tracts with decreased European ancestry in the same region. Furthermore, this region includes SNPs significantly associated with *AgeAccelHannum* in both published GWAS [32] and our dataset. Importantly, the alleles enriched in Native-American ancestry in the Criollos resulted to be associated with an increase in *AgeAccelHannum*. Overall, our data align with the general observation reported in a recent study on the *APOE* gene [42], where the authors demonstrated that the *APOEe4* risk allele is dependent on local ancestry. In particular, they showed that the ancestral background of a genomic region in which variants are located is crucial in conferring a diverse risk of Alzheimer’s disease, while the risk does not change when considering global ancestry [42]. The authors indeed reconstructed local ancestry patterns in individuals from African American and Puerto Rican populations, revealing that *APOEe4* alleles located in an African background conferred a lower risk than those with a European ancestral background, irrespective of the population.

Finally, to explore the different roles of genomic history and environmental factors (such as place of living and T. cruzi infection) in influencing the observed variability of epigenetic estimators, we employed the LGM model to measure the relative importance of predictors. The model highlighted that the variability of two out of eight epigenetic estimators (*AgeAccelGrim* and *DNAmB2MAdjAge*) is mostly influenced by genetic factors related to population ancestry, while the variability of three out of eight epigenetic estimators (*AgeAccelPheno*, *DNAmCystatinCAdjAge*, *DNAmTIMP1AdjAge*) is more affected by environmental factors, such as the place of living and the infection by T. cruzi. In this study, we selected Wichì and Criollos individuals with the same percentage of positive serology for T. cruzi (as outlined in ‘Methodology’ section), but their place of living differed, with Criollos individuals originating from urban area and the majority of Wichí (20 out of 24) instead coming from rural areas. As discussed above, the place of living is undoubtedly one of the main factors to consider in driving some of the differences observed. Interestingly, the infection by T. cruzi was found to impact *DNAmCystatinCAdjAge* in Criollos (but not in Wichí), with infected individuals displaying higher values compared to not-infected ones. It is important to highlight that *DNAmCystatinCAdjAge*, along with the other DNAm surrogate biomarkers, showed only a moderate correlation with protein blood levels in a previous study [7], thus warranting further investigations for a more comprehensive interpretation of this observation.

Overall, the relatively small percentage of variance generally explained by some of the observed predictors, however, reveals that additional unobserved variables, both genetic and non-genetic, may exert a significant influence on the variability of considered epigenetic estimators. This further highlights the importance of conducting additional studies on these populations.

## LIMITATIONS

Below are listed the main limitations of the present study:

- The present study considered a small sample size and more data are needed to validate our findings. However, when possible, we followed an extremely cautious approach in setting the study design by first screening previous GWAS data performed on larger cohorts and then conducting a replication analysis based on our dataset.
- The dataset we analyzed lacks biomedical information on the examined individuals and does not include an accurate characterization of some non-genetic factors that could potentially influence the observed differences. However, we collected population-based information from anthropological fieldwork to explore possible explanations for the differences observed.
- The epigenetic estimators used were created on multiple datasets, which, however, primarily included individuals of European ancestry. Despite their validation in many other populations of different ancestries, it cannot be completely ruled out that the predictions in indigenous populations may be affected by the composition of the selected training datasets. This further emphasizes the necessity of additional studies involving populations of non-European ancestry and the importance of incorporating the theme of human natural variation into the debate on epigenetic clocks, and more generally in the broader field of human aging.

## CONCLUSIONS

The present study describes the first-time the differences in epigenetic estimators between two human populations (Wichí and Criollos) with different ancestry and evolutionary genetic histories living in the same area of Gran Chaco in Argentina, by using an innovative integrated approach. Our findings demonstrate that Native-American Wichí exhibit a significant increase in epigenetic age compared to admixed Criollos. Furthermore, we show that the study of microevolutionary processes can yield important insights into the impact of past population dynamics on epigenetic estimators. The observed epigenetic differences between Wichí and Criollos cannot be attributed to a general association with the global ancestry. Instead, we suggested that microevolutionary forces may influence the genomic background of these populations, and this, in turn, may confer to individual chromosomal regions a different impact in terms of association with epigenetic clocks. Importantly, similar phenomena have been previously described in other studies examining other age-related traits [42]. However, we also demonstrate that microevolutionary processes alone cannot fully account for the observed differences in epigenetic age estimates between these two populations. In conclusion, our results highlight the crucial role of non-genetic factors, such as ecological conditions, nutrition, infectious diseases and socio-economic structures, in shaping the observed epigenetic variation.

## Supplementary Material

eoad034_suppl_Supplementary_Figures_S1

eoad034_suppl_Supplementary_Figures_S2

eoad034_suppl_Supplementary_File_S1

eoad034_suppl_Supplementary_File_S2

eoad034_suppl_Supplementary_File_S3

eoad034_suppl_Supplementary_Tables

## Data Availability

The DNA methylation data generated in the current study and used to calculate epigenetic clocks is available at 10.6084/m9.figshare.23736828. Other data are available upon request to the corresponding author.
