# Supplementary figures and images for: Epigenetic aging differences between Wichí and Criollos from Argentina: Insights from genomic history and ecology
Source: Evol Med Public Health. 2023 Oct 16;11(1):397–414. doi: 10.1093/emph/eoad034 (PMC10632719; doi:10.1093/emph/eoad034)

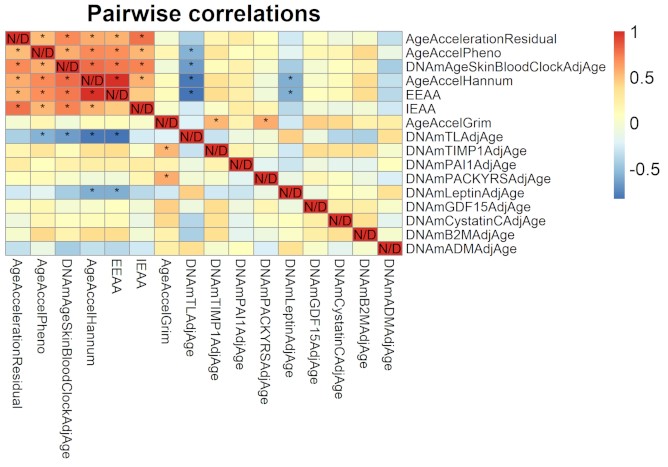

Supplement: eoad034_suppl_Supplementary_Figures_S1 [file eoad034_suppl_supplementary_figures_s1.jpeg]

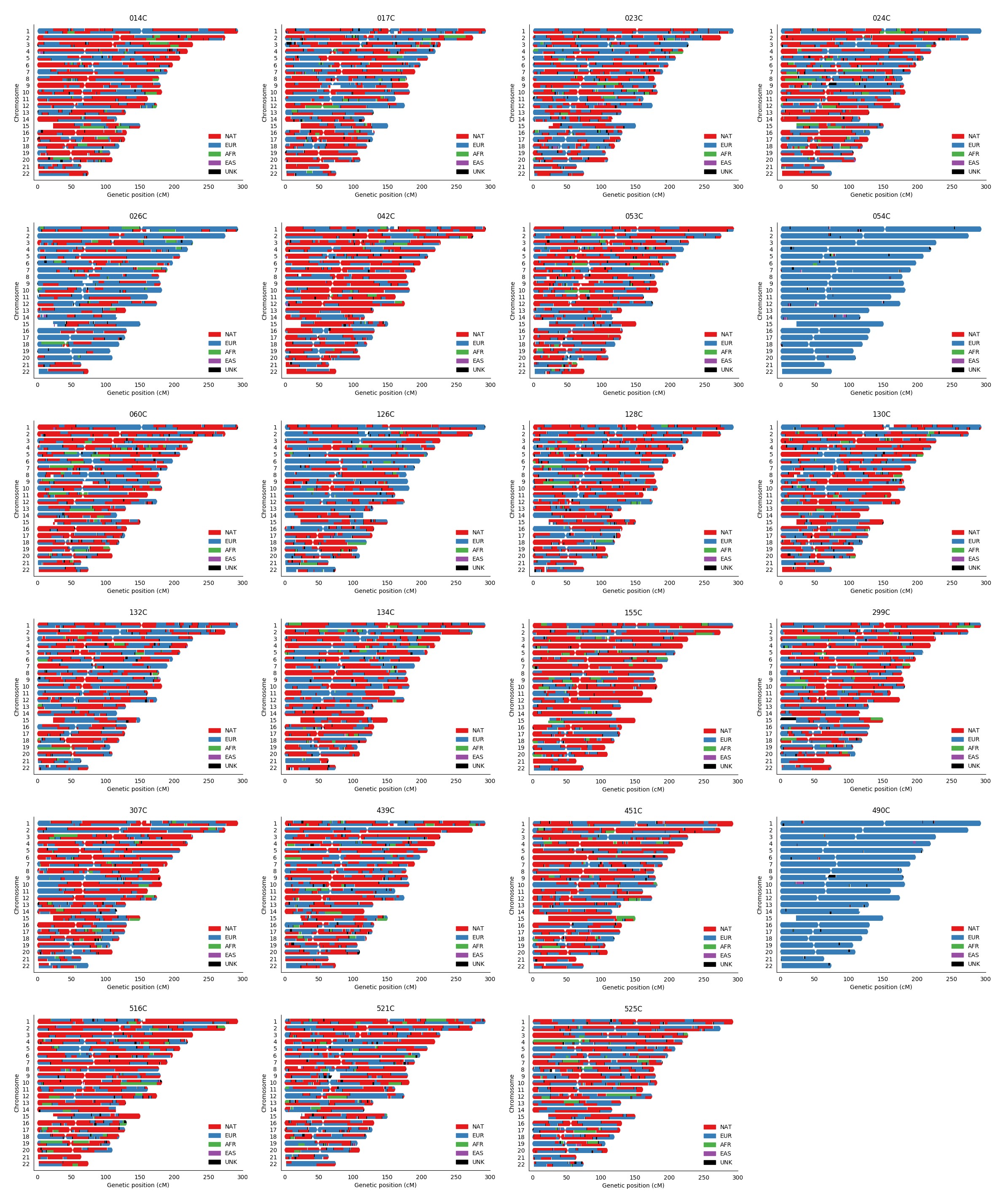

Supplement: eoad034_suppl_Supplementary_Figures_S2 [file eoad034_suppl_supplementary_figures_s2.jpeg]
