# Supplementary material for: Epigenetic aging differences between Wichí and Criollos from Argentina: Insights from genomic history and ecology
Source: Evol Med Public Health. 2023 Oct 16;11(1):397–414. doi: 10.1093/emph/eoad034 (PMC10632719; doi:10.1093/emph/eoad034)
Supplement: eoad034_suppl_Supplementary_File_S1 [file eoad034_suppl_supplementary_file_s1.docx]

**Supplementary File 1**

**Validation of DNA methylation age estimates and comparison of the pace of aging in Wichí and Criollos**

Among the several epigenetic estimators returned by the online DNAm age calculator ([http://dnamage.genetics.ucla.edu](http://dnamage.genetics.ucla.edu/)) implemented by Horvath [1], we considered and validated only the DNA methylation (DNAm) age estimators for further analysis: *DNAmAgeHorvath*, *DNAmAgeHannum*, *DNAmPhenoAge*, *DNAmGrimAge*, *DNAmAgeSkinBloodClock* [1–4] (see Table 1 for a detailed description).

| **DNAm age estimator** | **Description** |
| --- | --- |
| DNAmAgeHorvath | DNAm age estimate based on methylation of 353 CpG sites [1] |
| DNAmAgeHannum | DNAm age estimate based on methylation of 71 CpG sites [2] |
| DNAmPhenoAge | DNAm age estimate based on methylation of 513 CpG sites [3] |
| DNAmGrimAge | DNAm age estimate based on methylation of 1,030 CpG sites  build on eight DNAm based measures (DNAmADM, DNAmB2M, DNAmCystatinC, DNAmGDF15, DNAmLeptin, DNAmPACKYRS, DNAmPAI1, DNAmTIMP1) [4] |
| DNAmAgeSkinBloodClock | DNAm age estimate based on methylation of 391 CpG sites for human fibroblasts, keratinocytes, buccal cells, endothelial cells, lymphoblastoid cells, skin, blood, and saliva samples [1] |

**Table 1.** List of DNAm age estimator used to estimate the chronological ages of Wichí and Criollos

The accuracy of five DNAm age estimators (Table 1) was validated by calculating the Pearson coefficient (*r*) between each of them and the chronological age in Wichí and Criollos separately.

We consistently found that the DNAm age estimates were very accurate for Criollos (*r >* 0.9 for all the considered estimators) with a median absolute difference (MAD) between the true and the estimated ages that ranges from a minimum of 2.64 years, observed for the *DNAmPhenoAge* estimator, to a maximum of 4.97 years observed for the *DNAmAgeHorvath* estimator (Figure 2).

The correlation values observed in Wichí were instead generally lower, except for *DNAmGrimAge,* which showed a correlation value close to 1 (*r=0.97*) (Figure 2). The MAD ranges from a minimum of 2.26 years, observed for the *DNAmGrimAge* estimator, to a maximum of 6.33 years observed for the *DNAmAgeHorvath* estimator (Figure 2).

The correlation value observed in Wichí for the *DNAmAgeHorvath* estimator ($r_{DNAmAgeHorvath}$*=0.71*) is lower than the values observed for the population of European ancestry (which in general show correlation values greater than 0.9) [1] as it is the case with other native population previously published, such as the African rainforest hunter-gatherers and farmers ($r_{DNAmAgeHorvath}$*=0.84*) [5] or the Baka of the western Central African rainforest ($r_{DNAmAgeHorvath}$*=0.81*) [6]. Furthermore, we found that the discrepancy observed between the true and the estimated ages is lower in Wichí $({MAD}_{DNAmAgeHorvath}=6.33)$ than in Baka $({MAD}_{DNAmAgeHorvath}=13.06)$ [6].


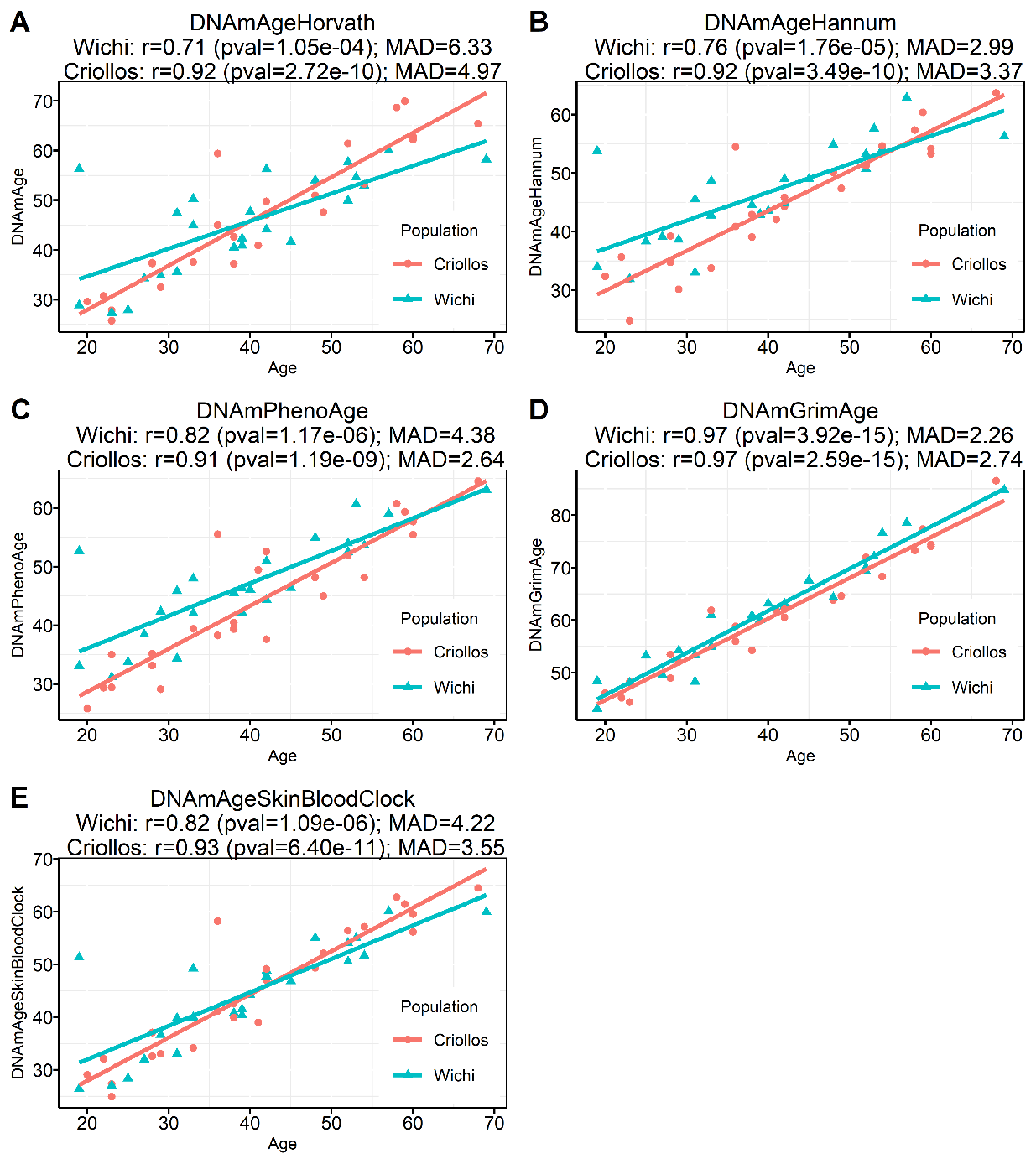


**Figure 2**. Scatterplots of chronological age against DNAm age estimates as predicted by the *Horvath* model (A), *Hannum* model (B), *PhenoAge* model (C), *GrimAge* model (D) and *SkinBloodClock* model (E). Pearson correlation coefficients ($r$), the related p-values (*pval*) and the median absolute differences (MAD) are reported for Wichí and Criollos separately.

In order to assess if the two population have different pace of aging (POA), we estimated a regression model for each of the five DNAm Age estimator as follow:

$$DNAmAge Estimator = \alpha+ \beta*Age+\gamma*Pop + \delta*(Age*Pop)$$

where $Age$ is the chronological age and $Pop$ is the belonging population. The regression coefficient $\gamma$ indicates the effect size of the predictor $Pop$ on the the DNAm age estimators, and in particular a value of $\gamma>0$ indicates that the DNAm Age estimates are higher in Wichí than in Criollos. The regression coefficient $\delta$ indicates the difference in the pace of aging (POA) between the two populations, and in particular a value of $\delta>0$ indicates that the POA is higher in Wichí than in Criollos. We found that Wichí have higher DNAm Age estimates than Criollos according to *DNAmAgeHorvath*, *DNAmAgeHannum*, and *DNAmAgePheno* (p-value < 0.05), whereas the POA did not differ significantly between the two population, except for the *DNAmAgeHorvath* estimator (p-value < 0.05) (Table 2).

| **DNAm age estimator** | **Effect size Age (** $\beta$ **)** | **P-value** $\beta$ | **Effect size population (** $\gamma$ **)** | **P-value** $\gamma$ | **Delta POA (**$\boldsymbol{\delta}$ **)** | **P-value** $\boldsymbol{\delta}$ |
| --- | --- | --- | --- | --- | --- | --- |
| DNAmAgeHorvath | 0.89 | 6.44e-12 | 13.43 | 0.03 | -0.33 | 0.02 |
| DNAmAgeHannum | 0.68 | 4.94e-12 | 11.19 | 0.02 | -0.20 | 0.07 |
| DNAmPhenoAge | 0.73 | 1.35e-12 | 11.01 | 0.02 | -0.18 | 0.11 |
| DNAmGrimAge | 0.77 | 2.00e-16 | 0.47 | 0.85 | 0.03 | 0.66 |
| DNAmAgeSkinBloodClock | 2.81 | 2.38e-13 | 7.75 | 0.12 | -0.18 | 0.12 |

**Table 2.** Summary statistics of the DNAm age estimators considered in this study. “**Effect size Age**” reports the variation in the DNAm Age estimates for an extra year of chronological age for both populations “**Effect size population**” reports the variation in the DNAm Age estimates for Wichí population; “**Delta POA**” reports the differences in the pace of aging between Wichí and Criollos.

Although these results suggest that Wichí are epigenetically older according to 3 out of 5 considered DNAm Age estimators, we cannot exclude a possible bias due to the lower accuracy of the above mentioned DNAm Age estimators in predicting chronological age in the Wichí whole blood methylation data set.

**References**

1. Horvath S. DNA methylation age of human tissues and cell types. *Genome Biology* 2013;**14**:R115.

2. Hannum G, Guinney J, Zhao L *et al.* Genome-wide Methylation Profiles Reveal Quantitative Views of Human Aging Rates. *Molecular Cell* 2013;**49**:359–67.

3. Levine ME, Lu AT, Quach A *et al.* An epigenetic biomarker of aging for lifespan and healthspan. *Aging* 2018;**10**:573–91.

4. Lu AT, Quach A, Wilson JG *et al.* DNA methylation GrimAge strongly predicts lifespan and healthspan. *Aging* 2019;**11**:303–27.

5. Fagny M, Patin E, MacIsaac JL *et al.* The epigenomic landscape of African rainforest hunter-gatherers and farmers. *Nat Commun* 2015;**6**:10047.

6. Gopalan S, Carja O, Fagny M *et al.* Trends in DNA Methylation with Age Replicate Across Diverse Human Populations. *Genetics* 2017;**206**:1659–74.
