# Supplementary material for: Epigenetic aging differences between Wichí and Criollos from Argentina: Insights from genomic history and ecology
Source: Evol Med Public Health. 2023 Oct 16;11(1):397–414. doi: 10.1093/emph/eoad034 (PMC10632719; doi:10.1093/emph/eoad034)
Supplement: eoad034_suppl_Supplementary_File_S2 [file eoad034_suppl_supplementary_file_s2.docx]

**Supplementary File 2**

**Analysis based on the infection by Tripanosoma cruzi**

Although having different genomic histories, Wichí and Criollos share the same natural environment since both live in the village of Mision Nueva Pompeya, located in the middle of the Gran Chaco region in Argentina.

In this area, Chagas disease is endemic without differences in susceptibility to Trypanosoma cruzi (T. cruzi) infection among the two groups. However, some differences in the long-term effects of the infection at cardiac level have been described and Wichí showed a lower prevalence of electrocardiographic abnormalities than Criollos (i.e. RBBB, right bundle branch block) [1].

Data about the 24 Wichí (mean age: 39.2 ± 12.9 yo, sex ratio 1:1) and 24 Criollos (mean age: 41.1 ± 14.03 yo, sex ratio 1:1) include information about infection by T. cruzi. In particular, the 50% (12/24) of Wichí and the 50% (12/24) of Criollos are infected by T. cruzi. The infection by T. cruzi has been measured by a serological test performed in 2008 and 2010. Whole blood samples were taken from venous blood.

Here we performed a T-test and Wilcoxon test to identify if epigenetic estimators are different in individuals infected or not by T. cruzi, for Wichí and Criollos separately. We found that Criollos individuals infected by T. cruzi showed slightly higher values of *DNAmCystatinCAdjAge* (nominal p-value=3.87e-02) than not infected, but we did not find any significant results in Wichí (Fig. 1).

*DNAmCystatinCAdjAge* is a DNAm-based surrogate marker and represents an estimator of plasma levels of cystatin C. It is used in the computation of the epigenetic clock GrimAge [2]. This finding supports the line of evidence suggesting that Native groups are more adapted to the ancestral environment and that they were evolutionarily selected to respond to local disease and pathogens [3]. Indeed, T. cruzi was well established at the time that the earliest human groups (members of the Chinchorro culture) first populated this segment of the Andean coast and they encountered mammal species that hosted this parasite [4]. In fact, Wichí were shown to belong to the same ancestral non-Andean lineages of all Amazonians that participated in the processes that have characterized the first peopling of South America [5], likely encountering the T. cruzi at the time of the first settling in this area.


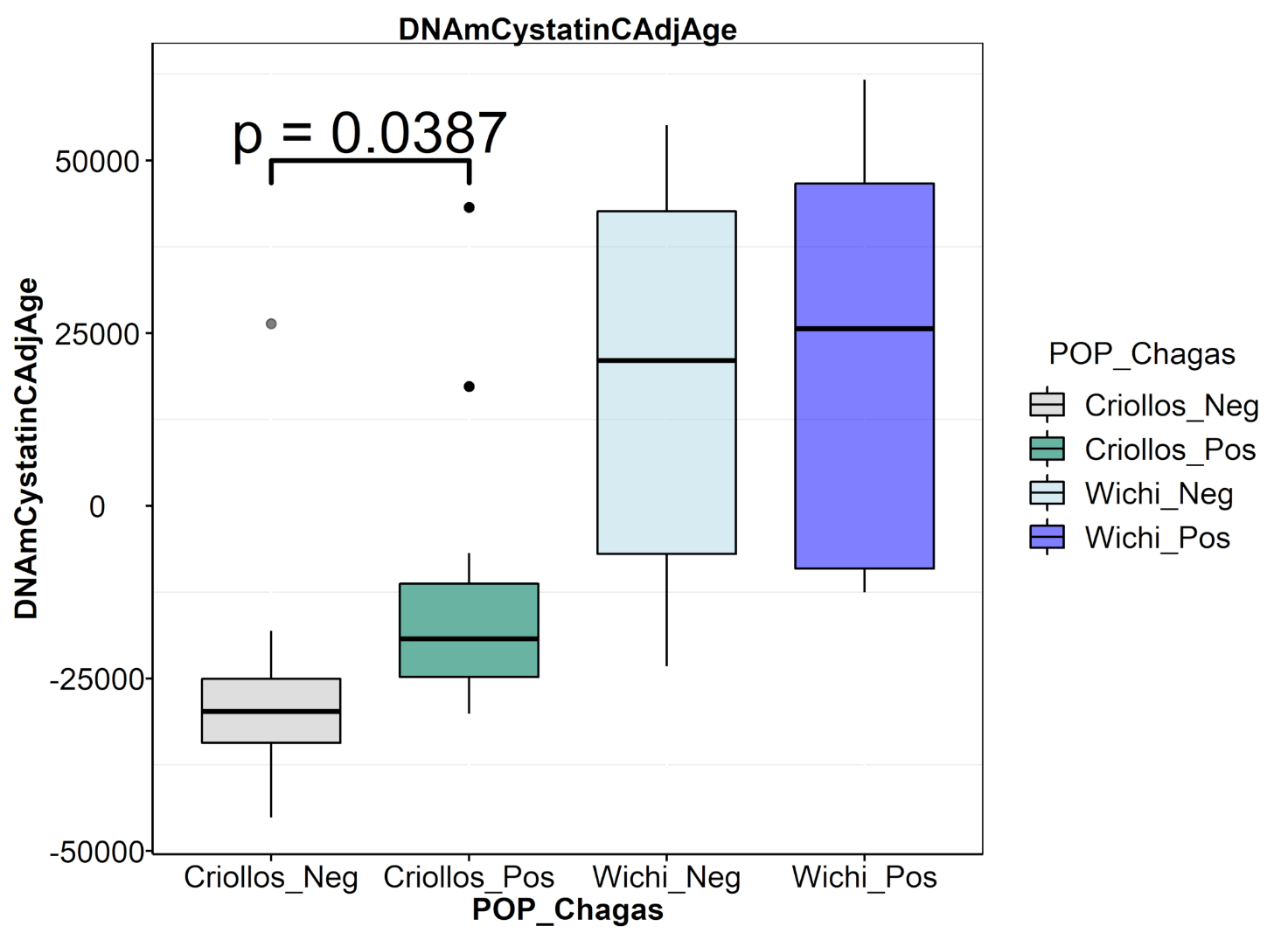


**Figure 1.** Boxplot for DNAmCystatinCAdjAge in Wichí and Criollos. “*Criollos_Neg*”: Criollos not infected by T. cruzi; “*Criollos_Pos*”: Criollos infected by T. cruzi; “*Wichí_Neg*”: Wichí not infected by T. cruzi; “*Wichí_Pos*”: Wichí infected by T. cruzi.

**References**

1. Moretti E, Castro I, Franceschi C *et al.* Chagas disease: serological and electrocardiographic studies in Wichi and Creole communities of Misión Nueva Pompeya, Chaco, Argentina. *Mem Inst Oswaldo Cruz* 2010;**105**:621–7.

2. Hillary RF, Stevenson AJ, Cox SR *et al.* An epigenetic predictor of death captures multi-modal measures of brain health. *Mol Psychiatry* 2021;**26**:3806–16.

3. Ojeda-Granados C, Abondio P, Setti A *et al.* Dietary, Cultural, and Pathogens-Related Selective Pressures Shaped Differential Adaptive Evolution among Native Mexican Populations. Gojobori DJ (ed.). *Molecular Biology and Evolution* 2022;**39**:msab290.

4. Aufderheide AC, Salo W, Madden M *et al.* A 9,000-year record of Chagas’ disease. *Proc Natl Acad Sci USA* 2004;**101**:2034–9.

5. Gnecchi-Ruscone GA, Sarno S, De Fanti S *et al.* Dissecting the Pre-Columbian Genomic Ancestry of Native Americans along the Andes–Amazonia Divide. Mulligan C (ed.). *Molecular Biology and Evolution* 2019;**36**:1254–69.
