# Supplementary material for: Epigenetic aging differences between Wichí and Criollos from Argentina: Insights from genomic history and ecology
Source: Evol Med Public Health. 2023 Oct 16;11(1):397–414. doi: 10.1093/emph/eoad034 (PMC10632719; doi:10.1093/emph/eoad034)
Supplement: eoad034_suppl_Supplementary_File_S3 [file eoad034_suppl_supplementary_file_s3.docx]

**Supplementary File 3**

**Epigenetic estimators and correlation with ancestry proportions in Criollos**

Global ancestry proportions were inferred via ADMIXTURE [1] by testing two through twelve genetic components (Fig. 1) and the best predictive accuracy according to CV errors was achieved when eight ancestral groups (K = 8) were hypothesized (Fig. 2).


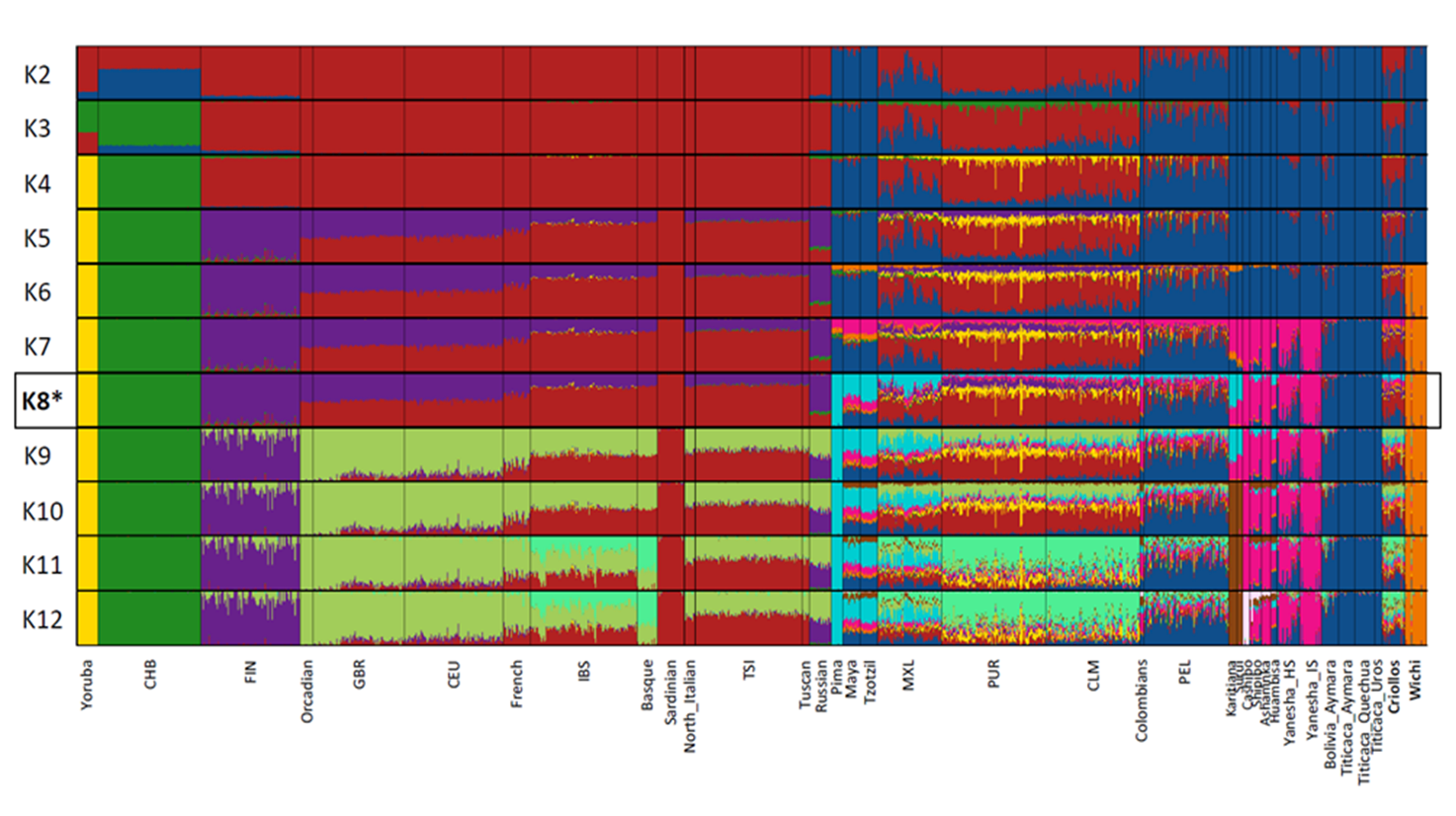


**Figure 1**. ADMIXTURE analysis performed on the 1,349 individuals from 36 populations of the extended dataset. At any K, from 2 (top) through 12 (bottom), each individual is represented by a vertical (100%) column of genetic component probabilities, colored according to the K reconstructed ancestral populations. Individuals are grouped and labeled at the population level. The best predictive model at K = 8 (which is plotted in more details on Fig. 3A) is highlighted


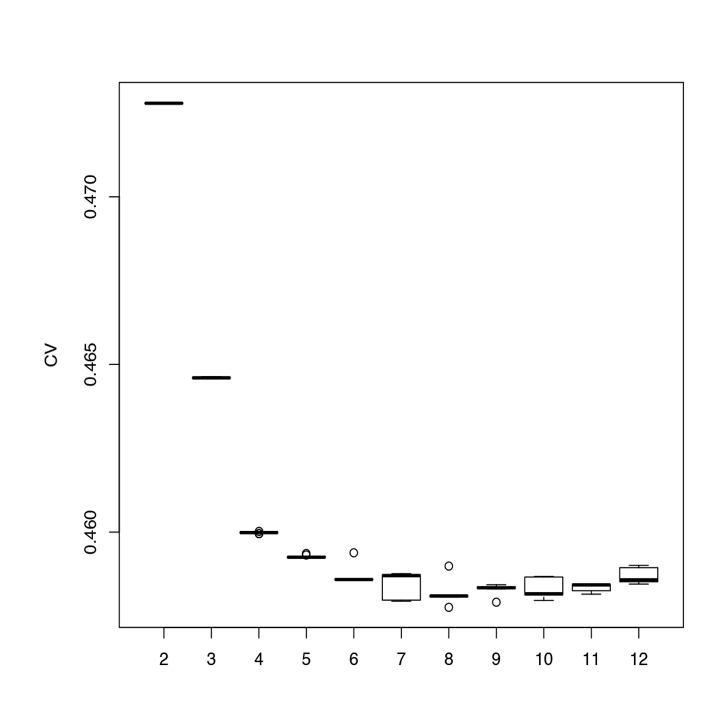


**Figure 2.** Boxplot of cross-validation errors for the ADMIXTURE runs in 10 replicates at K from 2 to 12. The best fitting model of admixture indicated by the minimum CV errors is K=8.

The major continental ancestry fractions were identified at K = 4 (Fig. 3A), where the main Native American, European, African and Asian genetic components are distinguished. At this level, the Wichí population presents on average more than 95% of Native-American ancestry (with 16 individuals having proportions > 99.9%). On the contrary, almost all Criollos show relatively even levels of Native American and European ancestries (on average 53.2% and 42.3% respectively), the only exception being two individuals that revealed extremely high European ancestry proportions. Contributions from the African and Asian components appeared instead generally low in both Wichí (0.33% and 0.16%) and Criollos (3.14% and 0.95%), respectively.

At the best predictive value of K = 8 (Fig. 3A), ADMIXTURE analysis confirmed the results from previous studies [2] by identifying highly-specific Native American genetic ancestry fractions. In particular, one component resulted specifically related to the Wichí population (here K8_1), one was highly represented in Mexican groups and Mesoamerican populations (K8_2), while other two appeared differentiated between Amazonian-specific (K8_4) and Andean-specific (K8_7) ancestries. The remaining components further reflected genetic ancestries related to Northern Europe (K8_3) and Southern Europe (K8_5) respectively, along with the major African (K8_6) and East Asian (K8_8) components. In this context, the Wichí consistently appeared almost entirely characterized by the Wichí-specific genetic component, while Criollos confirmed the observed levels of admixture, involving Native American- and European-related ancestries.


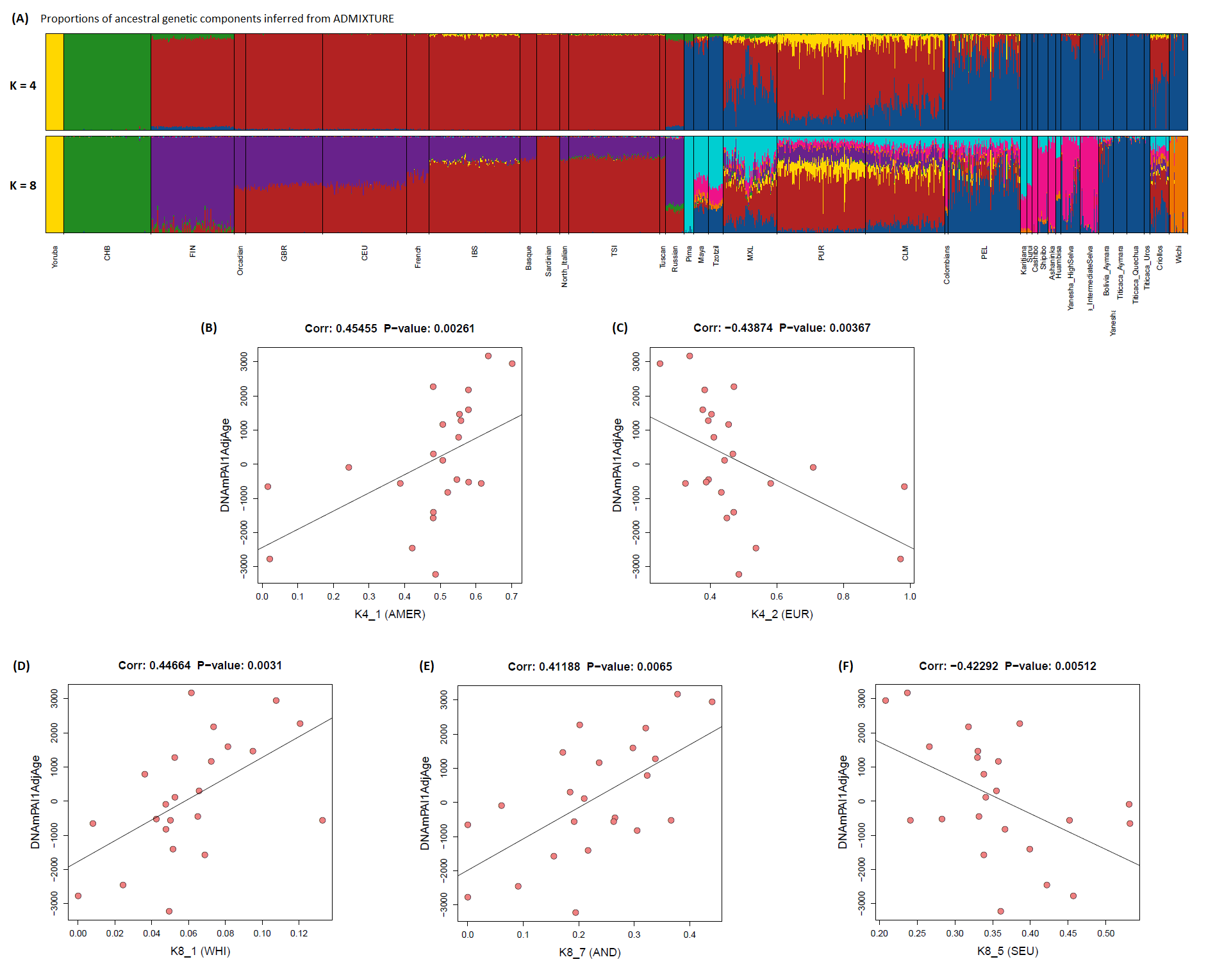


**Figure 3**. Population admixture and correlation with epigenetic estimators. (A) Global ancestry proportions estimated by ADMIXTURE clustering analysis at K = 4 and K = 8. Each individual is represented by a vertical (100%) column of genetic component probabilities, colored according to the K reconstructed ancestral populations. Individuals are grouped and labeled at the population level. Wichí and Criollos newly-analysed in the present study are highlighted in bold. Scatterplots of correlations between admixture genetic components (x-axis) and epigenetic estimators (y-axis) that resulted significant in the Criollos population are reported for the genetic ancestries inferred at both K = 4 (B-C) and K = 8 (D-F).

We then tested the correlation between genetic ancestry components and epigenetic estimators in the admixed Criollos group (Fig. 3B - 3F). At the continental level of admixture (K = 4), *DNAmPAI1AdjAge* was negatively associated to the European component (r2 = -0.43; p-value 3.67e-03) and positively associated to the Native American genetic ancestry (r2 = 0.45; p-value = 2.61e-03). These results were still significant after multiple test corrections (FDR < 0.05). The same signals remained significant also when eight ancestral groups were considered based on the best admixture CV value. Accordingly, in the admixed population of Criollos at K = 8, the Wichí (K8_1) and Andean-specific (K8_7) Native American genetic ancestries were associated to higher levels of *DNAmPAI1AdjAge* (r2 = 0.44, p-value = 3.10e-03 and r2 = 0.41, p-value = 6.50e-03, respectively), while the Southern European component (K8_5) was associated to the opposite effect, i.e*.* a decreased value of *DNAmPAI1AdjAge* (r2 = -0.42; p-value 5.12e-03). However only the association between *DNAmPAI1AdjAge* and K8_1 and the association between *DNAmPAI1AdjAge* and K8_5 remained significant after multiple test correction (FDR < 0.05).

Furthermore, the possible association of continental admixture and inbreeding was evaluated in Criollos by correlating the four Native American, European, Asian and African ancestry fractions with the inbreeding coefficients, but no significant result (p-value < 0.01) was observed.

The Criollos ancestry proportions estimated globally through ADMIXTURE at the same K = 4 continental-level of admixture were then correlated with the ancestry estimates obtained from the local inference procedure. The local ancestry components were inferred with RFmix [3] using four putative source groups informative of the main Native American, European, African and Asian continental genetic components. Correlation results particularly showed high and significant Spearman correlation coefficients (Native American ancestry: rho = 0.98, p-value = 1.93e-15; European ancestry: rho = 0.99, p-value < 2.20e-16; African ancestry: rho = 0.92, p-value = 1.46e-09), thus confirming the high concordance between the results obtained from the two methods. Correlation test was not performed for the Asian ancestry since it was consistently found at almost null frequency (<1%) in all Criollos individuals.

**References**

1. Alexander DH, Novembre J, Lange K. Fast model-based estimation of ancestry in unrelated individuals. *Genome Res* 2009;**19**:1655–64.

2. Gnecchi-Ruscone GA, Sarno S, De Fanti S *et al.* Dissecting the Pre-Columbian Genomic Ancestry of Native Americans along the Andes–Amazonia Divide. Mulligan C (ed.). *Molecular Biology and Evolution* 2019;**36**:1254–69.

3. Maples BK, Gravel S, Kenny EE *et al.* RFMix: A Discriminative Modeling Approach for Rapid and Robust Local-Ancestry Inference. *The American Journal of Human Genetics* 2013;**93**:278–88.
